# Phylogenomics of SAR116 Clade Reveals Two Subclades with Different Evolutionary Trajectories and an Important Role in the Ocean Sulfur Cycle

**DOI:** 10.1128/mSystems.00944-21

**Published:** 2021-10-05

**Authors:** Juan J. Roda-Garcia, Jose M. Haro-Moreno, Lukas A. Huschet, Francisco Rodriguez-Valera, Mario López-Pérez

**Affiliations:** a Evolutionary Genomics Group, División de Microbiología, Universidad Miguel Hernández, Alicante, Spain; b Research Center for Molecular Mechanisms of Aging and Age-related Diseases, Moscow Institute of Physics and Technology, Dolgoprudny, Russia; University of Hawaii at Manoa

**Keywords:** DMSP, population genomics, SAR116, marine *Alphaproteobacteria*, streamlined genomes, sulfur cycle

## Abstract

The SAR116 clade within the class *Alphaproteobacteria* represents one of the most abundant groups of heterotrophic bacteria inhabiting the surface of the ocean. The small number of cultured representatives of SAR116 (only two to date) is a major bottleneck that has prevented an in-depth study at the genomic level to understand the relationship between genome diversity and its role in the marine environment. In this study, we use all publicly available genomes to provide a genomic overview of the phylogeny, metabolism, and biogeography within the SAR116 clade. This increased genomic diversity has led to the discovery of two subclades that, despite coexisting in the same environment, display different properties in their genomic makeup. One represents a novel subclade for which no pure cultures have been isolated and is composed mainly of single-amplified genomes (SAGs). Genomes within this subclade showed convergent evolutionary trajectories with more streamlined features, such as low GC content (ca. 30%), short intergenic spacers (<22 bp), and strong purifying selection (low ratio of nonsynonymous to synonymous polymorphisms [*dN*/*dS*]). Besides, they were more abundant in metagenomic databases recruiting at the deep chlorophyll maximum. Less abundant and restricted to the upper photic layers of the global ocean, the other subclade of SAR116, enriched in metagenome-assembled genomes (MAGs), included the only two pure cultures. Genomic analysis suggested that both clades have a significant role in the sulfur cycle with differences in the way both clades can metabolize dimethylsulfoniopropionate (DMSP).

**IMPORTANCE** The SAR116 clade of *Alphaproteobacteria* is a ubiquitous group of heterotrophic bacteria inhabiting the surface of the ocean, but the information about their ecology and population genomic diversity is scarce due to the difficulty of getting pure culture isolates. The combination of single-cell genomics and metagenomics has become an alternative approach to study these kinds of microbes. Our results expand the understanding of the genomic diversity, distribution, and lifestyles within this clade and provide evidence of different evolutionary trajectories in the genomic makeup of the two subclades that could serve to illustrate how evolutionary pressure can drive different adaptations to the same environment. Therefore, the SAR116 clade represents an ideal model organism for the study of the evolutionary streamlining of genomes in microbes that have relatively close relatedness to each other.

## INTRODUCTION

Marine bacterioplankton play a central role in the sustainability of marine environments driving biogeochemical processes as well as primary production at the base of the food chain (1). Phytoplankton are believed to be responsible for approximately half of the total global primary production (1). In the microbial loop, heterotrophic bacteria are responsible for the assimilation and metabolization of labile dissolved organic matter (DOM) released by photoautotrophs in the aquatic environment (2–4). Variations in the availability and type of nutrients in the pelagic habitat have led to the emergence of distinct trophic strategies, oligotrophs and copiotrophs. Although it is difficult to reach a strict consensus on the defining characteristics of either group (5), oligotrophs are slow-growing bacteria highly adapted for optimal growth in nutrient-poor environments. In contrast, copiotrophic bacteria are characterized by their ability to grow under high nutrient concentrations, responding rapidly to nutritional changes in the environment (5–7). Some models for heterotrophic marine bacteria such as *Alteromonas* (8, 9), *Vibrio* (10), or *Roseobacter* (11) are copiotrophs. In offshore oligotrophic pelagic habitats, copiotrophic bacteria are minorities, and only the transient nutrients discharged from particulate organic matter, e.g., in algal blooms or animal ejecta, provide opportunities for their swift growth (12). However, in nutrient-enriched zones such as coastal waters or estuaries, these microbes play an important role in the ecosystem (13, 14). Molecular approaches targeting the 16S rRNA gene, such as fluorescence *in situ* hybridization (FISH), terminal restriction fragment length polymorphism (T-RFLP), and denaturing gradient gel electrophoresis (DGGE) and later the advent of next-generation DNA sequencing technologies, i.e., metagenomics, have proven that the surface ocean microbiome is mostly dominated by oligotrophs (15–19). Despite their abundance and importance, the bottleneck of acquiring pure cultures by classical culture-based approaches has considerably impeded their study. Thus, most of our present knowledge about these largely unknown but essential components of the biosphere and the ocean microbial ecosystem has been derived from metagenomics and single-cell genomics approaches (18–22). Most of the ocean water column, in contrast to soil, sediments, or animal bodies, is oligotrophic, i.e., containing highly diluted organic and inorganic nutrients. The microbes that thrive there are mostly oligotrophs that utilize nutrients in very low concentrations. For that, they need to keep a low surface-to-volume ratio, which translates into very small cells (23–25) (e.g., “*Candidatus* Pelagibacter ubique” has a 0.12- to 0.20-μm diameter and a cell volume of only 0.01 μm^3^ [26]). This minimization of cell size and complexity is coupled with highly compacted genomes characterized by (i) significant reduction in genome size with highly conserved core genomes and few pseudogenes, (ii) short intergenic spacers, (iii) low numbers of paralogs, and (iv) low GC content. These genomic features described as an evolutionary adaptation for more efficient use of nutrients in oligotrophic environments removing nonessential genes are referred to as “streamlining theory” (25).

Although underrepresented in comparison to these streamlined dominant groups such as the alphaproteobacterial SAR11 clade and the cyanobacterium *Prochlorococcus* (25), there are many other cosmopolitan lineages of heterotrophic marine bacterioplankton in the global oceans, including SAR116 and SAR86 clades within *Proteobacteria* or the *Actinomarinales* within the *Actinobacteria* (27, 28). Despite playing a central role in the function of marine ecosystems, they have received much less attention largely because only a few isolates have been isolated or characterized (29), and most of our knowledge about their ecological and genomic role comes from either metagenome-assembled genomes (MAGs) or single-cell genomes (SAGs).

Here, we applied an ecogenomic approach to 185 genomes of the SAR116 clade (*Alphaproteobacteria*), a ubiquitous group of heterotrophic bacteria inhabiting the surface of the ocean, to assess their potential role in the marine pelagic habitat (30). Their relative abundance based on 16S rRNA gene clone libraries varied in the range of 1% to 17% (29). To date, only two representatives of SAR116 have been cultured and their genomes sequenced, “*Ca.* Puniceispirillum marinum” IMCC1322 isolated from surface seawater of the East Sea Basin of Korea (31) and HIMB100, collected off the coast of Hawaii in the subtropical Pacific Ocean (32). Analysis of the genomes revealed common metabolic features including genes such as those for proteorhodopsins, carotenoid biosynthesis, and carbon monoxide dehydrogenase. In addition, the IMCC1322 strain plays an important role in the dimethylsulfoniopropionate (DMSP) cycle via the cleavage pathway to generate dimethylsulfide (DMS) in the surface waters of the oligotrophic ocean (33). The ocean represents a major reservoir of sulfur (mainly in the form of sulfates) on Earth (34). DMSP is an organosulfur compound produced by phytoplankton as compatible solute (35), which when degraded liberates gaseous DMS, one of the main sources of sulfur in the atmosphere and reduced sulfur as well as acrylate (36, 37).

Although several metagenomic studies of marine samples have obtained MAGs from this group (21, 38–40), recently their number has increased by ca. 100 new genomes coming from a large library of planktonic bacterial and archaeal SAGs collected from tropical and subtropical epipelagic ecosystems (22). This study has revealed a new perspective on the genomic complexity of the marine microbiome (22). The increased genomic diversity within this group has led to the discovery of two subclades of SAR116, which coexist in the same environment but appear to be subjected to different evolutionary pressures in their genomic makeup. The new subclade that emerged from the improved phylogenomic classification showed genomic features similar to streamlined genomes without genome size reduction. Despite genomic differences, metabolic reconstruction revealed a photoheterotrophic lifestyle with several genes involved in the metabolism of inorganic and organic sulfur compounds. We detected genes for the oxidation of sulfite and thiosulfate in both SAR116 subclades. In addition, we found marked differences in the degradation of the organic DMSP; while the isolate genomes and their closest relatives rely on DMSP lyase, the novel subclade contained exclusively genes involved in the demethylation pathway which produces (methylsulfanyl)propanoate (MMPA). Our data suggest that SAR116 might play a key role in the sulfur cycle in the surface ocean.

## RESULTS AND DISCUSSION

### Phylogenomic characterization of the SAR116 clade.

**FIG 1:**
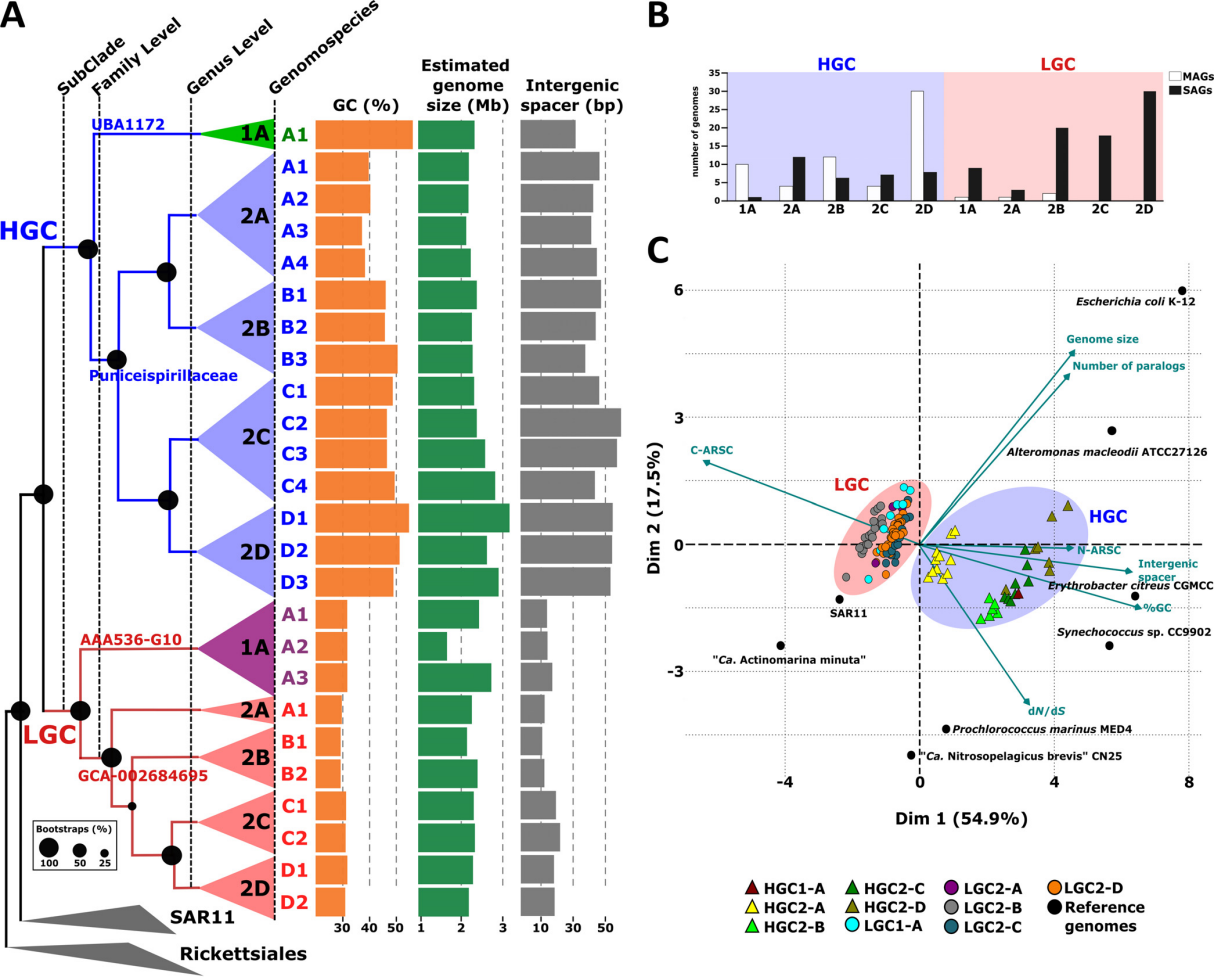
(A) Phylogenomic analysis of all SAR116 genomes available using a total of 258 concatenated conserved proteins to generate a maximum likelihood tree. The branches have been colored according to the subclade to which they belong (blue, High GC [HGC], and red, Low GC [LGC]). The genomes of nearby orders SAR11 and *Rickettsiales* were used as outgroup. GC content, together with estimated genome size and intergenic spacer, is plotted next to the tree. (B) Number of SAGs and MAGs belonging to each genus within the HGC and LGC subclades. (C) Principal-component analysis (PCA) was performed using several genomic parameters: *dN*/*dS*, GC content, intergenic spacer, estimated genome size, N-ARSC, and C-ARSC as well as the number of paralogous genes found in the genomes of the HGC and LGC subclades in comparison with several reference genomes.

A total of 185 genomes were downloaded from publicly available databases putatively classified as members of the SAR116 clade (based on NCBI classification accessed in August 2020; see Materials and Methods), which includes only two cultured representatives (IMCC1322 and HIMB100) together with 120 SAGs and 63 MAGs that met the established quality criteria of ≥50% completeness and ≤5% contamination, i.e., medium- to high-quality draft genomes (41) (see Table S1 in the supplemental material). Phylogenomic analysis using a concatenation of 258 single-copy marker proteins showed that SAR116 genomes clustered into two subclades with four different families (two per subclade) (Fig. 1A and Fig. S1). Based on GTDB classification (42), these four families were placed within the *Puniceispirillales* order (Table S1). The two pure culture representatives were placed in the same family (*Puniceispirillaceae*) that together with family UBA1172 clustered within one of the subclades characterized by containing a higher proportion of MAGs (59 MAGs and 43 SAGs) (Fig. 1A and B and Fig. S1). On the other hand, the other subclade, composed of families AAA536-G10 and GCA-002684696, was represented mostly by SAGs (*n* = 86) including only 4 MAGs (Fig. 1A and B and Fig. S1). Most of these SAGs (79 of the 86 genomes) come from a large collection of genomes sampled from the surface (epipelagic) ocean in tropical and subtropical latitudes (22) (Table S1). Therefore, this intrinsic difficulty in obtaining pure cultures and in reconstructing genomes from metagenomes of this new subclade has kept its genomic diversity hidden until now with the advance in single-cell genomics. Clustering based on pairwise average nucleotide identity (ANI) (Fig. S2) revealed groups of genomes within each family with ANI values of ca. 70%, which placed these strains likely as different genera, named A to D for simplicity (Fig. 1A). In the end, we were able to distinguish two subclades, four families, and 10 putative genera within the SAR116 clade (Fig. 1A, Fig. S1, and Table S1).

10.1128/mSystems.00944-21.1 FIG S1 Maximum likelihood phylogenomic tree of all SAR116 genomes available. The branches have been colored according to the subclade to which they belong (blue, High GC [HGC], and red, Low GC [LGC]). Genera within each family are separated by lines and named on the outermost part. Genomes were grouped within genomospecies (black or white circles). Download FIG S1, PDF file, 0.08 MB. Copyright © 2021 Roda-Garcia et al. 2021 Roda-Garcia et al. https://creativecommons.org/licenses/by/4.0/ This content is distributed under the terms of the Creative Commons Attribution 4.0 International license.

10.1128/mSystems.00944-21.2 FIG S2 Pairwise comparison among the SAR116 genomes using average nucleotide identity (ANI). Rectangles delimit subclades and genera. Download FIG S2, PDF file, 0.3 MB. Copyright © 2021 Roda-Garcia et al. 2021 Roda-Garcia et al. https://creativecommons.org/licenses/by/4.0/ This content is distributed under the terms of the Creative Commons Attribution 4.0 International license.

10.1128/mSystems.00944-21.5 TABLE S1 Detailed information about the genomes used in this study. Download Table S1, XLSX file, 0.03 MB. Copyright © 2021 Roda-Garcia et al. 2021 Roda-Garcia et al. https://creativecommons.org/licenses/by/4.0/ This content is distributed under the terms of the Creative Commons Attribution 4.0 International license.

### Differential genomic features of the SAR116 subclades.

Once the phylogenomic classification of the whole clade was established, genomic features were evaluated for each group. To be as precise as possible, we have used only genomes from single-cell sequencing. We calculated the GC content (%GC), intergenic spacer length, and the estimated genome size (Fig. 1A and Table S1). Interestingly, we found a significant variation of the GC content between the two subclades. While the subclade containing pure culture representatives (*Puniceispirillaceae* and UBA1172 families) showed a wide range of values from 37.91 to 51.39%, (mean subclade 45.10% [standard deviation {SD}, ±5.87]), GC content value was consistent across all genera in the new subclade (30.55 ± 0.87) (Fig. 1A and Tables S2 and S3). Based on these significant differences (*P* value <0.01), we named the two subclades High GC (HGC) and Low GC (LGC) (Fig. 1A). Lower GC content has been suggested to be an adaptation in nitrogen-limited environments such as open ocean regions (25). In fact, we observed changes in the amino acid usage between the two groups. The LGC subclade showed higher prevalence for basic amino acids such as asparagine and lysine with only one N atom in side chains. However, members of the HGC group had a higher frequency of arginine (3 N in side chain) (Table S4). Another useful approach to examine the overall encoded nitrogen and carbon content is the average number of nitrogen or carbon atoms per amino acid residue side chain (N-ARSC and C-ARSC, respectively). Significant differences were found for both parameters between the two groups. We found that the LGC group had a lower nitrogen content in amino acid residue side chains (0.331 ± 0.003 versus 0.336 ± 0.004; *P* value <0.01) and a higher C-ARCS (3.08 ± 0.02 versus 2.91 ± 0.06; *P* value <0.01) (Tables S2 and S3). This same correlation between low GC content and low N-ARSC has already been reported in other groups such as *Marinimicrobia* (43). In these microbes, groups inhabiting nutrient-poor waters showed a decrease in the N-ARSC of the proteins encoded in comparison with their mesopelagic counterparts. However, our genomes come from the same environment, which might suggest specific adaptations to microniches such as planktonic or particle association (44).

10.1128/mSystems.00944-21.6 TABLE S2 Genomic features of the SAR116 subclades. Download Table S2, XLSX file, 0.01 MB. Copyright © 2021 Roda-Garcia et al. 2021 Roda-Garcia et al. https://creativecommons.org/licenses/by/4.0/ This content is distributed under the terms of the Creative Commons Attribution 4.0 International license.

10.1128/mSystems.00944-21.7 TABLE S3 Genomic features of the SAR116 genomes genus versus reference genomes. Download Table S3, XLSX file, 0.01 MB. Copyright © 2021 Roda-Garcia et al. 2021 Roda-Garcia et al. https://creativecommons.org/licenses/by/4.0/ This content is distributed under the terms of the Creative Commons Attribution 4.0 International license.

10.1128/mSystems.00944-21.8 TABLE S4 Comparison of amino acid usage in SAR116 genera. Download Table S4, XLSX file, 0.01 MB. Copyright © 2021 Roda-Garcia et al. 2021 Roda-Garcia et al. https://creativecommons.org/licenses/by/4.0/ This content is distributed under the terms of the Creative Commons Attribution 4.0 International license.

In addition to the GC content, we observed a significant variation in the intergenic spacer length (Tables S2 and S3). While in HGC the average length between genes was between 35 and 57.69 bp (mean subclade 50.94 bp [SD, ±7.53]), for all of the genera of the LGC subclade median spacers were <25 bp (mean subclade 18.36 bp [SD, ±4.71]), with values as low as 9 bp in the case of LGC2-B (Fig. 1A and Tables S1, S2, and S3). Although the estimated genome size was also statistically significant (*P* value 0.01) between the two subclades, the difference in mean values was not as divergent as for the other parameters (Tables S2 and S3). Among all the genera, the estimated genome size was ca. 2.4 Mb, with the only exception of the genus HGC2-D, which showed a genome size higher than the rest with an average of 3.23 Mb (SD, ±0.43) (Fig. 1A and Tables S1, S2, and S3). Likewise, this genus also exhibited high values for both GC content and intergenic spacer sizes. As a consequence of the smaller size of the intergenic space, genomes within LGC had higher numbers of genes per megabase of genome (1,036 [SD, ±35] versus 963 [SD, ±20]; *P* value <0.01) (Tables S2 and S3).

These genomic features suggested that members within the LGC subclade are experiencing a streamlining process. For that reason, we studied other characteristic genomic parameters that have been proposed to be relevant in the streamlined genomes such as selective pressure and the number of paralogs (43, 45–47). Microevolution was measured as the ratio of nonsynonymous to synonymous polymorphisms (*dN*/*dS* ratio). We found that the median *dN*/*dS* value was 0.09 (SD, ±0.02) for LGC; this value was comparable to the better-known marine SAR11 clade (45) and suggests a strong purifying selection acting on the genome evolution of this subclade (Tables S2 and S3). Within the HGC subclade, we observed much more variable values. While the genus HGC2-A showed similar values as LGC, 0.065 (SD, ±0.004), in the other genera within HGC we found markedly higher median *dN*/*dS* values (from 0.13 to 0.18) (Table S3). However, the number of paralogs was the only parameter that was not differential between the two subclades (*P* value 0.23) (Table S2).

To put these genomic features into perspective, we compared these groups with a collection of reference marine microbes with different ecological strategies (Fig. 1C and Table S3). Despite the divergence, genomes within the LGC subclade showed consistent genomic parameters, some of them (GC content and *dN*/*dS* ratio) typical of well-studied streamlined genomes such as SAR11 or “*Ca*. Actinomarina minuta” (46) (Fig. 1C and Table S3). The median intergenic distance was higher than that of these two microbes, although it was slightly lower than that for other marine microbes with streamlined genomes such as the marine ammonia-oxidizing thaumarchaeon “*Ca.* Nitrosopelagicus brevis” CN25 and the cyanobacterium Prochlorococcus marinus CCMP1986 (Fig. 1C and Table S3), and the estimated genome size was double that of all these four reference genomes (SAR11, “*Ca*. Actinomarina minuta,” *P. marinus*, and “*Ca.* Nitrosopelagicus brevis”). The C-ARSC was greater than three for all genomes within the LGC group, similarly to all streamlined reference genomes. We found a negative linear correlation between C-ARSC and GC content (*R*^2^ 0.936, data not shown). However, for N-ARSC the correlation was positive but the coefficient was very low (*R*^2^ 0.315, data not shown). The HGC group shows multiple genomic evolutionary trajectories with features more similar to marine copiotrophic heterotrophs such as *Erythrobacter* and *Alteromonas* or the cyanobacterium *Synechococcus* sp. strain CC9902. The case of the HGC2-A group is outstanding in displaying an intermediate trend with strong purifying selection and lower GC more similar to LGC (Fig. 1C and Table S3). In addition, like LGC groups, HGC2-A had a higher proportion of genomes recovered by single-cell genomics (Fig. 1B).

### Ecological distribution (metagenomic recruitment).

**FIG 2:**
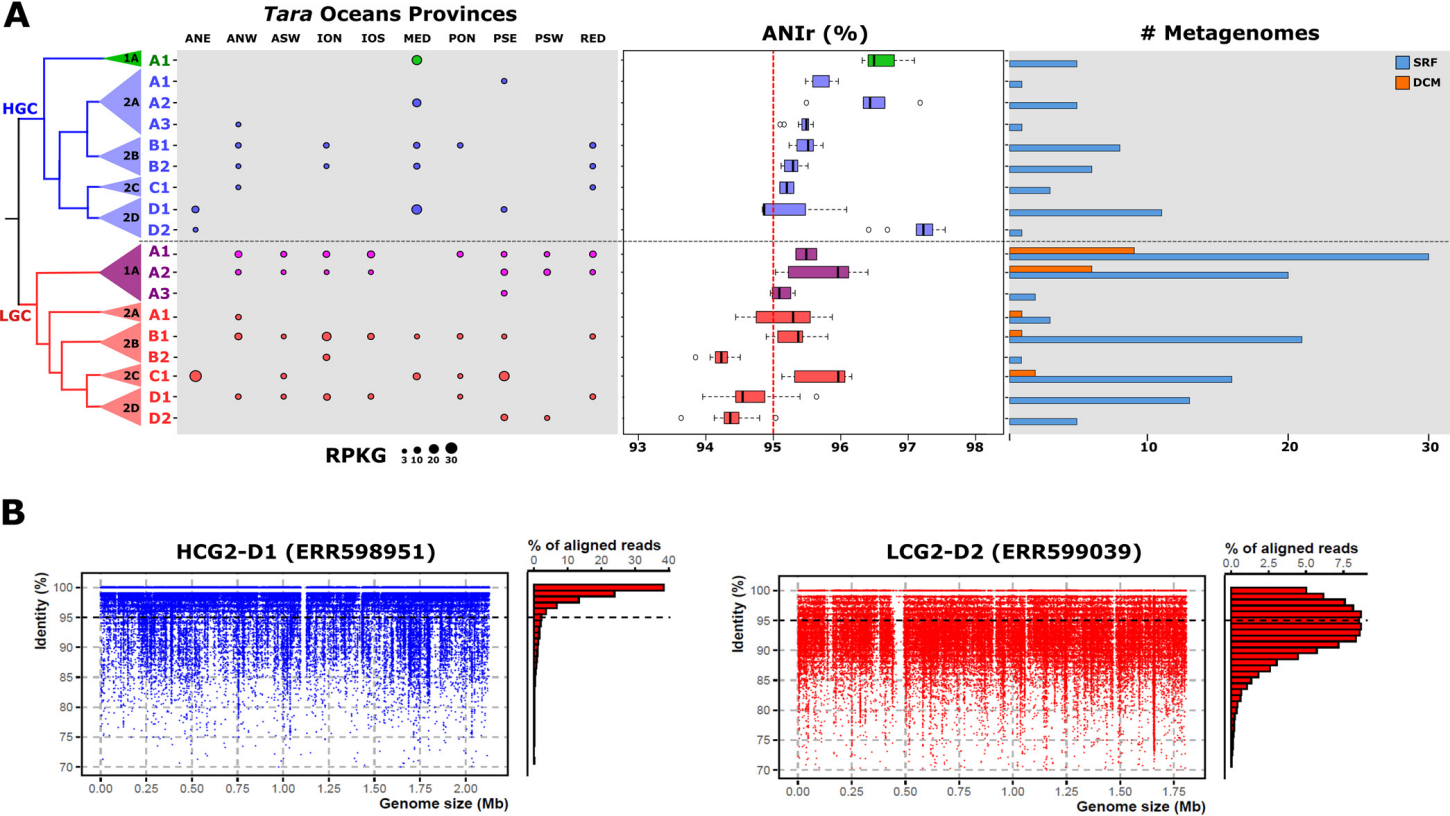
(A) Relative abundance (measured in RPKG) of SAR116 genomospecies in *Tara* Ocean metagenomes. Box plot in the middle indicates the average nucleotide identity based on metagenomic reads (ANIr) among SAR116 genomospecies. Occurrence of SAR116 genomes within *Tara* stations is shown on the right. Bars indicate the number of metagenomic samples where genomes recruit at least three RPKG (presence). A maximum likelihood phylogenomic tree of the SAR116 clade is shown on the left. Box plots and dots from the recruitment were colored according to the different families following the patterns in Fig. 1. (B) Linear recruitment plot of the representative genomes for HGC2-D1 and LGC2-D2 genera. Each blue dot represents a metagenomic read. The histogram on the right shows the relative percentage of aligned reads in intervals of 1% identity. The black dashed line indicates the species threshold (95%).

The differential genomic features observed between the two subclades could be related to adaptations to specific ecological niches. Therefore, we analyzed the distribution patterns using metagenomic read recruitment analysis in the large global data set from the *Tara* Oceans Project (20). First, we analyzed the relative abundance of the genomes (see Materials and Methods) against their occurrence in the metagenomic samples, which allowed for the determination of several genomospecies, i.e., groups of genomes with close phylogenomic relationship and similar relative abundances within the same geographical locations (46, 48). We were able to differentiate 23 genomospecies (Fig. 1A, Table S1, and Fig. S1). The minimum pairwise ANI value among these ecogenomic units of classification was ca. 85%. The results showed that SAR116 microbes were found exclusively associated with the upper layers of the epipelagic zone. None of the genomospecies was present in the cold-water stations of the Southern Ocean or mesopelagic zones (>200 m) (Fig. 2A). While HGC members were found only in surface waters, LGCs showed a broader distribution, present at a higher number of stations and depths, which suggests adaptation to a wider range of conditions (Fig. 2A). For instance, genomospecies LGC1-A1 and LGC1-A2 recruited in the highest number of stations from surface and deep chlorophyll-maximum (DCM) (Fig. 2A). While genomospecies HGC2-B1 and HGC2-B2 together with LGC1-A1, LGC1-A2, LGC2-B1, LGC2-C1, and LGC2-D1 could be considered the most cosmopolitan, present in several oceanic provinces from 30°N to 30°S, other genomospecies were restricted to specific regions such as the Mediterranean Sea (HGC1-A1 and HGC2-A2) and Pacific Ocean South East (HGC2-A1 and LGC1-A3) (Fig. 2A). The highest recruitment values (>20 reads per kilobase of genome and gigabase of metagenome [RPKGs]) within the HGC subclade corresponded to the HGC1-A1 and HGC2-D1 genomospecies at the same station in the eastern Mediterranean Sea (TARA_025). Regarding the other subclade, LGC2-C1 presented the highest recruitment values in station TARA_004 (ANE; Atlantic North East) together with TARA_094 and TARA_096 from temperate waters in the South Pacific Ocean (Fig. 2A).

In order to evaluate the intrapopulation sequence diversity, we used the metagenomic recruited reads to determine the read-based average nucleotide identity (ANIr). Most genomospecies in both subclades (HGC and LGC) showed a median ANIr value of ca. 95% (species threshold). None of the genomospecies within the HGC presented a lower value, but genomospecies HGC1-A1, HGC2-A2, and HGC2-D2 showed ANIr values of >96%, i.e., lower intrapopulation sequence diversity. These genomospecies could be considered endemic to the Mediterranean Sea and the station TARA_004 (located at the connection between the Mediterranean and the Atlantic Ocean). Therefore, it could suggest a more recent divergence of these groups adapted to the special conditions of the Mediterranean such as limiting P concentration. A similar example has already been described in the SAR11 genomospecies Ia.3/VII, which also showed a preferential presence in the Mediterranean (45). However, three LGC subclade genomospecies (LGC2-B2, LGC2-D1, and LGC2-D2) showed higher intrapopulation diversity, which could indicate higher ecological persistence over time of these populations (Fig. 2A) (49). This is reflected in the linear recruitment plots of these genomospecies (LGC2-D2) with a minimum alignment identity threshold of ca. 85% and HGC2-D1, whose pattern could be associated with a less diverse population (ca. 97%) (Fig. 2B).

The linear recruitments revealed the presence of metagenomic islands in two genomospecies (LGC1-A1 and LGC2-C1) belonging to different families within the LGC subclade in metagenomic samples from different locations (Fig. S3A and B). The results showed a highly hypervariable region that was always preserved in the same location among the genomes within the same genomospecies. Detailed analysis of the gene content showed that they are involved in synthesizing the outer glycosidic envelope of the cells (such as the O-chain polysaccharide in Gram-negative bacteria) (Fig. S3C). This high diversity found in this cell component has previously been explained because the components are important phage recognition targets (50). Since viruses identify their host through such exposed structures, the need to change the surface is obviously compelling. Thus, a good evolutionary strategy would be to vary these polysaccharides.

10.1128/mSystems.00944-21.3 FIG S3 (A and B) Linear recruitment plots of representative genomes of genomospecies LGC1-A1 (A) and LGC2-C1 (B) in two metagenomes. (C) An overview of the characteristic metabolism encoded in the flexible metagenomic island found in representatives within LGC2-C1. Download FIG S3, PDF file, 0.6 MB. Copyright © 2021 Roda-Garcia et al. 2021 Roda-Garcia et al. https://creativecommons.org/licenses/by/4.0/ This content is distributed under the terms of the Creative Commons Attribution 4.0 International license.

### General metabolic features within SAR116 HGC and LGC genomes.

**FIG 3:**
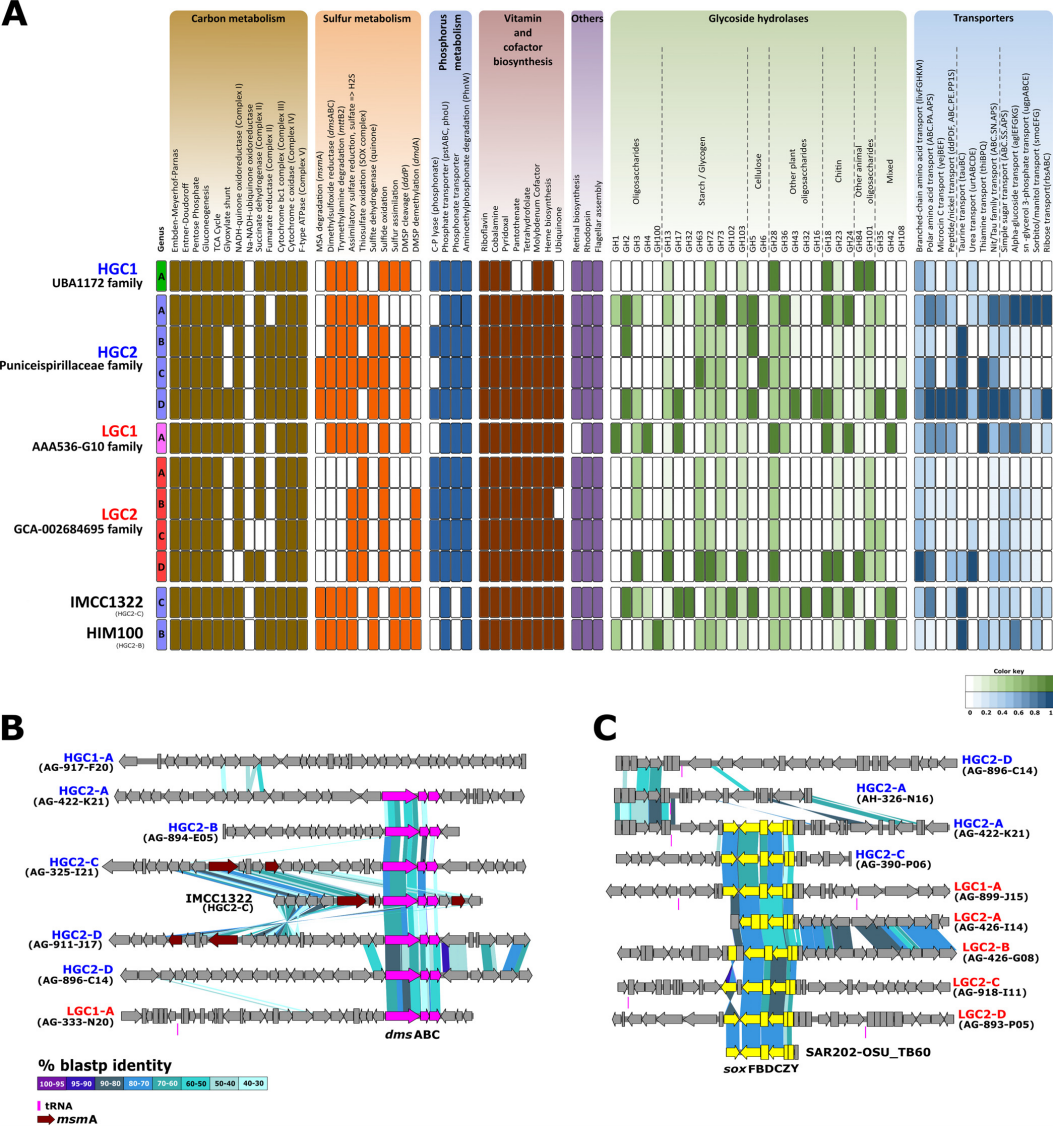
(A) Inferred metabolism of the 10 SAR116 genera (grouped by family) based on the KEGG database. “*Ca*. Puniceispirillum marinum” (IMCC1322) and alphaproteobacterium HIMB100 were added for the comparison. Modules within carbon, sulfur, and phosphate metabolism as well as vitamins and those determined as “others” were manually categorized as present/absent. For the GH and transporter categories, a range of values between 0 and 1 was established. The highest value found for each enzyme or transporter in a genus was determined as 1, and the rest of the values were normalized according to this value. (B) Genomic alignment (in amino acids) of the *dms*ABC and *msm*A genes found in SAR116 genomes. (C) Genomic alignment (in amino acids) of the *sox* operon found in SAR116 genomes. The fragment of SAR202-OSU_TB60 was added for the comparison as the closest relative.

The isolation and sequencing 1 decade ago of two strains, IMCC1322 and HIMB100 (31, 32), shed light on the physiology and metabolic potential of the SAR116 clade in the oceans. Here, with the increased genomic diversity of SAGs and MAGs, we have expanded the knowledge of this ubiquitous marine group. Given the incomplete nature of SAGs and MAGs, we clustered the genes of all the genomes belonging to each genus, and this clustering was used to analyze the metabolism against several functional databases (see Materials and Methods). For reference, we also included in the comparison the two pure culture genomes (HIMB100 and IMCC1322) that were classified into HGC2-B and HGC2-C, respectively (Table S1). Most of the results are in agreement with previous metabolic reports (31, 32) (Fig. 3A). Both HGC and LGC subclades are aerobic, chemoorganotrophic microorganisms, encoding enzymes for the tricarboxylic acid cycle (TCA cycle), and have the complexes I to IV involved in the electron transport chain (ETC). In addition, the three common glycolysis pathways (Embden-Meyerhof-Parnas, Entner-Doudoroff, and pentose phosphate) were also present in both subclades, although as reported from the pure cultures (31, 32), all genomes lack 6-phosphofructokinase (*pfk*A). However, in the ETC, some differences arose among subgroups. Complex II succinate dehydrogenase could not be detected within the genus LGC2-C (18 genomes).

The most common version of the complex I detected was the H^+^-NADH ubiquinone oxidoreductase (*nuo*) operon. This system was detected in the HGC subclade and the LGC1-A, LGC2-A, and LGC2-B genera. However, we detected that within the genomes of LGC2-C and LGC2-D the *nuo* operon was replaced with the sodium equivalent Na^+^-pumping NADH:quinone oxidoreductase (*nqr*) operon. Homology search against the nr NCBI database showed that the closest relative of this *nqr* operon was found with a low identity in the methylotrophic bacterium HTCC2181 (67.52% average amino acid identity) (Fig. S4A). It has already been reported that multiple horizontal gene transfer (HGT) events have allowed the dispersal of this operon among different bacterial lineages (51). In fact, we found a gene belonging to the *nuo* cluster (*nuo*L) in these genomes immediately adjacent to the *nqr* operon which is not present in the HTCC2181 genome (Fig. S4A). The use of sodium ion transport to generate an electrochemical potential that can be used both for ATP synthesis and as a primary sodium pump to maintain ionic homeostasis could be an evolutionary advantage in the marine environment. These replacements between the bioenergetic complexes (*nuo* and *nqr*) have already been reported in other marine bacterial lineages such as *Flavobacteria* (52) or members of the phylum *Marinimicrobia* where the presence of these different versions of respiratory complex I has been correlated with improved ecological adaptation to discrete niches (epipelagic and mesopelagic environments) (53). Likewise, Salcher et al. (54) described this change in bacteria of the *Methylophilaceae* family as an adaptation across the transition from freshwater to the marine environment.

10.1128/mSystems.00944-21.4 FIG S4 (A) Genomic alignment (in amino acids) of the different versions of the respiratory complex I (*nuo* and *nqr* operon) found in SAR116 genomes. *nqr* operon of *Methylophilales* was added for the comparison. (B) Comparison of the proteorhodopsin gene cluster that includes genes involved in the retinal synthesis. Download FIG S4, PDF file, 0.08 MB. Copyright © 2021 Roda-Garcia et al. 2021 Roda-Garcia et al. https://creativecommons.org/licenses/by/4.0/ This content is distributed under the terms of the Creative Commons Attribution 4.0 International license.

The glyoxylate shunt (GS), a two-step metabolic pathway that serves as an alternative to the TCA cycle, was detected only in some genera of the HGC subgroup (HGC1, HGC2-A, and HGC2-D) and LGC1. In addition, we detected marked differences in the acquisition and degradation of multiple sugar compounds. Overall, families of glycoside hydrolases (GHs) involved in the degradation of simple and complex oligosaccharides, such as glycogen, cellulose, or chitin, and sugar transporters were detected in all subgroups, although we found an enrichment of GH families within genera HGC2 and LGC1 (Fig. 3A). Contrastingly, the low numbers of these degradative enzymes within LGC2 and HGC1 may indicate different ecological strategies degrading organic carbon sources (e.g., cellulase was detected only in HGC2).

Regarding the metabolism of amino acids and vitamins, all genera of both subclades carried the necessary genes for biosynthesis of the 20 common amino acids (data not shown) and the vitamins B_2_ (riboflavin), B_5_ (pantothenate) B_6_ (pyridoxal), B_9_ (folate), and B_12_ (cobalamin), the molybdenum cofactor, and the heme group (Fig. 3A). Functional annotation of proteins indicated that instead of using the aspartate 4-decarboxylase, involved in the transformation of aspartate to alanine, they synthesize the latter via the enzyme 2-aminoethylphosphonate aminotransferase (*phn*W) from pyruvate and phosphonate (55, 56).

Lastly, we analyzed the presence of some ecologically relevant features. Most of the newly described genera, except LGC1, HGC2-A and HGC2-B, contained genes involved in the acquisition and degradation of phosphonates from seawater. Some regions, such as the Mediterranean or Sargasso Sea, are depleted in phosphate; organisms inhabiting these places need access to other P compounds (e.g., phosphonates) to grow and/or survive (48, 57). All genera of both subclades encoded the synthesis of a proteorhodopsin (58), and the amino acid sequence analysis indicated that all of them were proton pumps (DTE motif [59]) and most of them (90 out of 91) absorbed in the blue spectrum (60). Next to the proteorhodopsin (colocated on the same strand) is found the gene cluster involved in the synthesis of retinal (Fig. S4B). This cluster was present in all groups of both subclades except LGC1 (Fig. S4B). The position of these genes varies between HGC and LGC, and among genera within the HGC groups, which could suggest several independent acquisition events after a common ancestor (Fig. S4B). However, in all members of the LGC subclade, the gene coding for isopentenyl diphosphate isomerase (*isp*A) is not present. This genomic deletion forces the bacterium to retrieve retinal from the environment, like many other marine streamlined organisms (46, 61, 62). Despite the different evolutionary trajectories in terms of genomic architecture, at the functional level, the two subclades appear to have many similarities including the absence of essential genes in certain pathways, suggesting that multiple traits have been conserved through vertical inheritance.

### Contribution of SAR116 to the sulfur cycle in the ocean.

**FIG 4:**
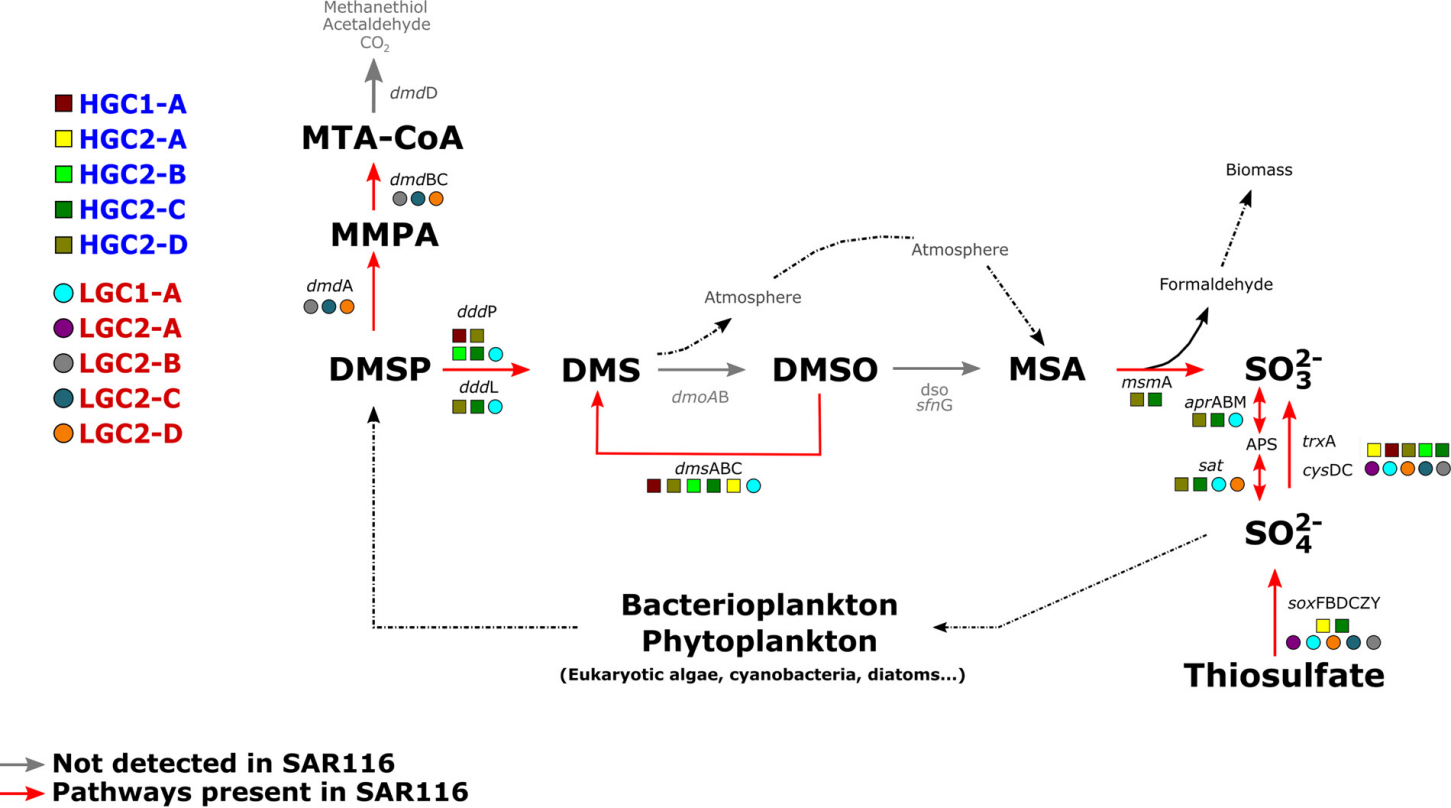
Representative view of the metabolic features found in the different genera of SAR116 related to sulfur cycling. The red lines show the pathways present. Circles and squares indicate genera within the LGC and HGC subclades, respectively.

Functional inference of SAR116 genomes showed that this clade plays a key role in the sulfur cycle (Fig. 4). We found two types of DMSP lyases, *ddd*L and *ddd*P (Fig. 4), that cleave DMSP to yield DMS. Then, DMS could be biotically oxidized to dimethyl sulfoxide (DMSO) by the enzyme DMS monooxygenase (*dmo*AB) or reduced again to DMS under anaerobic conditions (63) by the enzyme DMSO reductase (*dms*ABC) (Fig. 4). There is an alternative route to degrade DMSP, which involves the demethylation of DMSP to produce 3-(methylsulfanyl)propanoate (MMPA) by the activity of the enzyme dimethylsulfoniopropionate demethylase (*dmd*A). This is the first step to assimilate sulfur from DMSP into biomass. Some bacteria, such as Alteromonas macleodii and Ruegeria pomeroyi, can continue this pathway to produce acetaldehyde plus methanethiol (*dmd*BCD genes) (64).

Figure 4 shows a clear differentiation in the degradation of DMSP by the two SAR116 subclades. Genes involved in the generation of DMS (either through degradation of DMSP using DMSP lyases or by reduction from DMSO [*dms*ABC genes]) were detected only in the genomes of the HGC2 and LGC1 subgroups (Fig. 3B), while the demethylation pathway (*dmd*A) was exclusively detected on LGC2. Regarding the rest of the genes involved in the degradation of MMPA to methanethiol, we found homologs to *dmd*B and *dmd*C with low identity (ca. 40%), but not for *dmd*D. This same pattern has been described in SAR11, suggesting that the function of this gene (*dmd*D) could be replaced by other nonorthologous isofunctional enzymes (64). Remarkably, the main pathway to degrade DMSP, found in many epipelagic microorganisms (36), seems to be less relevant in the SAR116 clade. Previous reports indicated that this clade was the dominant dddP-containing bacterium in the Pacific Ocean (33). DMSO can be further metabolized to methanesulfonate (MSA), which is in turn cleaved to formaldehyde and sulfite by the methanesulfonate monooxygenase (*msm*A). We could identify the MsmA protein in the genomes HGC2-C and HGC2-D, in close proximity to the *dms*ABC gene cluster (Fig. 3B).

Lastly, the SAR116 clade contains several genes involved in sulfur oxidation systems, including the adenosine-5′-phosphosulfate reductase (*apr*ABM) and sulfate adenylyltransferase (*sat*) genes, which catalyze the oxidation of sulfite to sulfate, but only in the genera HGC2-C, HGC2-D, and LGC1 (LGC2D contains only the *sat* gene [Fig. 4]), as well as the oxidation of thiosulfate by the *sox* operon, widely distributed among LGC1 and LGC2 SAR116 groups, but also detected in HGC2-A and HGC2-C (Fig. 3C and Fig. 4). Previous studies demonstrated the presence and activity of sulfur-oxidizing chemolithoautotrophs to use reduced sources of sulfur (e.g., SUP05 and OM252 clades) in anaerobic waters (65, 66), but also in the photic aerobic water column in which *sox* genes are common (21, 67, 68), for energy generation, sometimes coupled to inorganic carbon fixation (69). In this sense, it seems that SAR116, like many other marine prokaryotes (70, 71), may be capable of generating energy from the oxidation of inorganic sulfur on surface waters. The LGC1 group despite its streamlined genome seems to have a higher metabolic versatility than the rest of the LGC group, more similar in this sense to the HGC members, not only in sulfur metabolism but also with a higher richness of both GHs and transporters (Fig. 3), which could be one of the reasons for its abundance at the DCM (Fig. 2).

### Conclusions.

In this study, we have characterized the members of the SAR116 clade, abundant marine heterotrophic bacteria. To date, the difficulty of obtaining large numbers of pure cultures using standard methods (to date there are only two pure cultures) and the scarcity and low reliability of MAGs have hindered our knowledge about their diversity, metabolic potential, and ecology. Now, the increase of databases with genomes from single-cell genomics has made it possible. Phylogenetic analysis suggests that this group of aerobic and chemoorganotrophic microorganisms consists at least of two subclades, four families, and 10 genera. A new subclade widely represented by SAGs showed genomic characteristics that indicate an evolutionary process of streamlining similar to other dominant marine microbes such as members of the alphaproteobacterial *Pelagibacterales* (SAR11 clade) and the “*Ca*. Actinomarinales” (46, 48, 72). According to this theory, which suggests that these modifications in the architecture of the genome represent an evolutionary advantage to oligotrophic environments, microbes from this new subclade (LGC) present a more cosmopolitan distribution compared to the other subclade.

Despite their genomic divergence, the high similarity within the LGC group in the genomic features analyzed suggests that these genomes have reached the limit of the process of genome streamlining. Except for the number of paralogous genes, all genomic parameters analyzed showed statistical significance between the two subclades, which provides a model for studying the evolutionary history of streamlined genomes. In the other subclade (HGC), there is a wide range of genomic architectures that may be due to different evolutionary histories or adaptations to different ecological niches. The presence of a genus (HGC2-A) with characteristics similar to those of LGC in terms of streamlining suggests that this evolutionary process may emerge in independent clades with parallel evolutionary trajectories. Although this study based on culture-independent approaches is a step further in understanding the population structure of this clade, genomic information obtained on the metabolic capabilities of these groups should be focused in future work on designing new isolation strategies not only to obtain more strains but also to understand their role in aquatic environments.

The example of SAR116 is not unique since other examples of processes with the same trend have already been described. In most of these cases, single-cell genomics is revealing new lineages of microbes with streamlined genomes that are very abundant in nature but difficult to obtain in pure culture (46, 48, 73). In the case of the marine *Roseobacter* clade, the use of single-cell genomics has allowed for the description of uncultivated streamlined lineages that together represent ca. 60% of the global pelagic *Roseobacter* bacteria in the ocean (74). Streamlined genomic features have been also linked to epipelagic *Marinimicrobia* compared with their mesopelagic counterparts (43) as well as an evolutionary response to the transition between different habitats in other bacterial groups (47, 54). The combined application of culture-independent approaches and single-cell genomics together with second- and recently third-generation sequencing to analyze the ocean microbiome will probably reveal other ecologically relevant clades. The systematic comparison of closely related streamlined and nonstreamlined lineages offers a unique opportunity for the study of similarities in evolutionary trajectories, as well as the possible role of the environment.

## MATERIALS AND METHODS

### Phylogenomic characterization.

All available genomes belonging to the SAR116 clade were downloaded from the National Center for Biotechnology Information (NCBI), based on the Genome Taxonomy Database (GTDB) (42) (available up to August 2020) (see Table S1 in the supplemental material). CheckM v1.1.3 using *lineage_wf* with default parameters (75) was used to estimate completeness and degree of contamination of the genomes, and only those with completeness of >50% and contamination of <5% were kept. Phylophlan3 was used to establish the phylogenomic classification with a total of 258 genes shared among all suitable genomes using the following parameters: *-d phylophlan -t a –diversity high –accurate -f supermatrix_aa.cfg* (76). We modified the program to use IQ-TREE (77) with LG+F+G4 amino acid model and an ultrafast bootstrap of 1,000 replicates (78). Along with the SAR116 genomes, a total of 85 reference genomes belonging to the SAR11 and *Rickettsiales* orders of the *Alphaproteobacteria* class were included as an outgroup. The resulting phylogenomic tree was analyzed and edited using iTOL (79).

### Genome comparison.

For each genome, coding DNA sequences (CDS) were predicted with Prodigal v2.6.3 using *-a output.proteins -d output.genes -c -p meta* parameters (80). These sequences were annotated against the NCBI database of nonredundant protein sequences (NCBI nr) using DIAMOND v2.0.6 (*blastp –sensitive –max-target-seqs 1 –evalue 1-e5 –block-size 12.0 –index-chunks 1*) (81) and against COG v2003 (update 2014) (82) and TIGFRAM v15.0 (September 2014) (83) using HMMscan v3.1b2 (84) *-E 1e-5 –notextw –noali* and default parameters. Subsequently, tRNAs were obtained using tRNAscan-SE v1.4 (85) and ssu-align v0.1.1 (86) along with meta-RNA (87) for rRNA genes. To establish similarity of the genomes, the ANI between all the genomes was calculated using the JSpecies v1.2.1 (88) package with standard parameters. Intrapopulation sequence diversity within each group was calculated using the average nucleotide identity of metagenomics read with the enveomics R package (89). To analyze streamlined genomic parameters, GC content was calculated using the gecee program from the EMBOSS package (90). For each genome, the number of paralogs was retrieved using CD-HIT v4.8.1, iterating from 90% to 30%, in steps of 20% identity (91) using the following parameters: *-c identity -G 0 -M 0 -T 0 -d 0 -aS 0.5 -p 1 -g 1 -sc 1*. Intergenic spacer size was calculated by measuring the distance between consecutive genes in all the genomes using an in-house perl script. N-ARSC and C-ARSC values for each gene were calculated using the script at https://github.com/faylward/pangenomics/blob/master/get_ARSC.py (92). As a reference, we have included in the comparison representatives of well-known microbes: *Pelagibacter* sp. HTCC7211 (NCBI accession number GCA_000155895.1) (93), “*Candidatus* Actinomarina” sp. AG-915-F11 (NCBI accession number GCA_902635395.1) (22), Alteromonas macleodii ATCC 27126 (NCBI accession number GCA_000172635.2) (94), Erythrobacter citreus LAMA-915 (NCBI accession number GCA_001235865.1) (95), *Synechococcus* sp. strain CC9902 (NCBI accession number GCA_000012505.1) (96), “*Ca*. Nitrosopelagicus brevis” CN25 (NCBI accession number GCA_000812185.1) (97), Prochlorococcus marinus MED4 (NCBI accession number GCA_000011465.1) (98), and Escherichia coli strain K-12 substrain MG1655 (NCBI accession number GCA_000005845.2) (99). The nonsynonymous (*dN*) and synonymous (*dS*) substitutions were computed for all orthologous genes between two entire genomes using the orthologr package (100). Based on the ANI values obtained, all genomes that fell within the species definition (ANI >95%) were selected for *dN*/*dS* analysis. Each genome was analyzed against the others in a pairwise comparison. The mean for all species was determined to obtain the *dN*/*dS* value for each genus. First, BLASTp was used to align and identify all orthologous sequences by choosing the best reciprocal hit, applying a threshold of >75% amino acid sequence identity and the pal2nal script (101) to perform codon alignment. Then, for each pair of sequences, *dN*, *dS*, and *dN*/*dS* ratios were computed based on the codon alignment using the YN method (102). We removed *dN*/*dS* values of ≥10 because they may already be due to methodological artifacts. *dN*/*dS* values of <1 indicate purifying selection, whereas higher values (*dN*/*dS* > 1) are a clear signal of diversifying selection (genetic drift). For statistical analysis, quantitative variables were expressed as the mean ± standard deviation (SD) and compared by the unpaired *t* test. Statistical analysis was performed using SPSS version 15.0 (SPSS, Inc., Chicago, IL). All *P* values were two-sided, and *P* < 0.05 was considered statistically significant. In order to compare the genomic features of the SAR116 genomes against several reference genomes, previously mentioned in this section, a principal-component analysis (PCA) was performed using several genomic parameters: *dN*/*dS*, GC content, intergenic spacer and estimated genome size, N-ARSC, and C-ARSC as well as the number of paralogous genes. The FactoMineR (103) and factoextra (https://github.com/kassambara/factoextra) libraries of R were used for this analysis. The FactoMineR library was used to standardize the data during the PCA. The plot was made using the Biplot function.

### Metagenomic fragment recruitment and SAR116 biogeography.

Metagenomes from the *Tara* Oceans expedition (20) were used to study ecological distribution patterns of SAR116 genomes. Metagenomic reads were aligned using BLASTn v2.10.1 (104). A cutoff of 98% nucleotide identity was established over a minimum alignment length of 50 nucleotides. To avoid possible bias due to the high potential for chimera generation in MAGs (41), we analyzed the relative abundance only of the data set of genomes that came from single-cell genomics and pure culture. Only those genomes recruiting at least three reads per kilobase of genome and gigabase of metagenome (RPKG) and with a genome coverage of ≥70% were kept for further analyses. In addition, in all genomes the rRNA operon was removed before recruitment to avoid the background noise it generates due to its high similarity between divergent genomes as previously reported (46, 48). The same parameters were used for the metagenomic linear recruitment. The resulting alignments, together with the distribution of the reads according to the identity of the alignment (histogram), were plotted using the ggplot2 package in R.

### Functional classification.

Since most of the genomes used are incomplete (MAGs and SAGs), we decided to cluster the collection of all gene sequences in all genomes belonging to the established genera to compare them at the functional level. Gene clusters were generated using CD-HIT v4.8.1 (91) with a minimum percentage of identity of 70%, as well as a coverage of at least 50%. The resulting gene clusters were annotated against three databases, SEED using DIAMOND v0.9.34 (81) (40% identity and coverage greater than 50%), CAZy (105) using dbCAN (106) (HMMER mode, E value 10^−15^ and coverage greater than 35%), and KEGG (107) (KEGG Mapper, Reconstruct Brite, KEGG Orthology) using the BlastKOALA V.2.2 tool (108). We added in the comparison the two pure culture genomes as a reference (IMCC1322 and HIMB100). All pathways within carbon, sulfur, and phosphate metabolism as well as vitamins and those determined as “others” were manually categorized as complete or not based on KEGG and MetaCyc results (109). For the GH and transporter categories, a range of values between 0 and 1 was established, with 1 being the maximum value for each category present in a genus and normalizing the value of the rest of the genera from that value. This was done independently for each enzyme or transporter found.

## References

[1] Field CB, Behrenfeld MJ, Randerson JT, Falkowski P. 1998. Primary production of the biosphere: integrating terrestrial and oceanic components. Science 281:237–240. doi:10.1126/science.281.5374.237. 9657713

[2] Buchan A, LeCleir GR, Gulvik CA, González JM. 2014. Master recyclers: features and functions of bacteria associated with phytoplankton blooms. Nat Rev Microbiol 12:686–698. doi:10.1038/nrmicro3326. 25134618

[3] Gómez-Pereira PR, Schüler M, Fuchs BM, Bennke C, Teeling H, Waldmann J, Richter M, Barbe V, Bataille E, Glöckner FO, Amann R. 2012. Genomic content of uncultured Bacteroidetes from contrasting oceanic provinces in the North Atlantic Ocean. Environ Microbiol 14:52–66. doi:10.1111/j.1462-2920.2011.02555.x. 21895912

[4] Arandia-Gorostidi N, Weber PK, Alonso-Sáez L, Morán XAG, Mayali X. 2017. Elevated temperature increases carbon and nitrogen fluxes between phytoplankton and heterotrophic bacteria through physical attachment. ISME J 11:641–650. doi:10.1038/ismej.2016.156. 27922602 PMC5322308

[5] Ho A, Di Lonardo DP, Bodelier PLE. 2017. Revisiting life strategy concepts in environmental microbial ecology. FEMS Microbiol Ecol 93 (3). doi:10.1093/femsec/fix006. 28115400

[6] Koch AL. 2001. Oligotrophs versus copiotrophs. Bioessays 23:657–661. doi:10.1002/bies.1091. 11462219

[7] Lauro FM, McDougald D, Thomas T, Williams TJ, Egan S, Rice S, DeMaere MZ, Ting L, Ertan H, Johnson J, Ferriera S, Lapidus A, Anderson I, Kyrpides N, Munk AC, Detter C, Han CS, Brown MV, Robb FT, Kjelleberg S, Cavicchioli R. 2009. The genomic basis of trophic strategy in marine bacteria. Proc Natl Acad Sci USA 106:15527–15533. doi:10.1073/pnas.0903507106. 19805210 PMC2739866

[8] López-Pérez M, Gonzaga A, Martin-Cuadrado A-BB, Onyshchenko O, Ghavidel A, Ghai R, Rodriguez-Valera F. 2012. Genomes of surface isolates of Alteromonas macleodii: the life of a widespread marine opportunistic copiotroph. Sci Rep 2:696. doi:10.1038/srep00696. 23019517 PMC3458243

[9] Azam F, Malfatti F. 2007. Microbial structuring of marine ecosystems. Nat Rev Microbiol 5:782–791. doi:10.1038/nrmicro1747. 17853906

[10] López-Pérez M, Jayakumar JM, Haro-Moreno JM, Zaragoza-Solas A, Reddi G, Rodriguez-Valera F, Shapiro OH, Alam M, Almagro-Moreno S. 2019. Evolutionary model of cluster divergence of the emergent marine pathogen vibrio vulnificus: from genotype to ecotype. mBio 10:e02852-18. doi:10.1128/mBio.02852-18. 30782660 PMC6381281

[11] Wagner-Döbler I, Biebl H. 2006. Environmental biology of the marine Roseobacter lneage. Annu Rev Microbiol 60:255–280. doi:10.1146/annurev.micro.60.080805.142115. 16719716

[12] Hou S, López-Pérez M, Pfreundt U, Belkin N, Stüber K, Huettel B, Reinhardt R, Berman-Frank I, Rodriguez-Valera F, Hess WR. 2018. Benefit from decline: the primary transcriptome of Alteromonas macleodii str. Te101 during Trichodesmium demise. ISME J 12:981–996. doi:10.1038/s41396-017-0034-4. 29335641 PMC5864184

[13] Williams TJ, Joux F, Lauro FM, Matallana-Surget S, Cavicchioli R. 2011. Physiology of marine oligotrophic ultramicrobacteria, p 1179–1199. In Horikoshi K (ed), Extremophiles handbook. Springer, Tokyo, Japan.

[14] Buchan A, Gonzalez JM, Moran MA. 2005. Overview of the marine Roseobacter lineage. Appl Environ Microbiol 71:5665–5677. doi:10.1128/AEM.71.10.5665-5677.2005. 16204474 PMC1265941

[15] Mullins TD, Britschgi TB, Krest RL, Giovannoni SJ. 1995. Genetic comparisons reveal the same unknown bacterial lineages in Atlantic and Pacific bacterioplankton communities. Limnol Oceanogr 40:148–158. doi:10.4319/lo.1995.40.1.0148.

[16] Giovannoni SJ, Britschgi TB, Moyer CL, Field KG. 1990. Genetic diversity in Sargasso Sea bacterioplankton. Nature 345:60–63. doi:10.1038/345060a0. 2330053

[17] Giovannoni SJ, Tripp HJ, Givan S, Podar M, Vergin KL, Baptista D, Bibbs L, Eads J, Richardson TH, Noordewier M, Rappé MS, Short JM, Carrington JC, Mathur EJ. 2005. Genome streamlining in a cosmopolitan oceanic bacterium. Science 309:1242–1245. doi:10.1126/science.1114057. 16109880

[18] Delong EF, Preston CM, Mincer T, Rich V, Hallam SJ, Frigaard N, Martinez A, Sullivan MB, Edwards R, Brito BR, Chisholm SW, Karl DM. 2006. Community genomics among microbial assemblages in the ocean’s interior. Science 311:496–503. doi:10.1126/science.1120250. 16439655

[19] Rusch DB, Halpern AL, Sutton G, Heidelberg KB, Williamson S, Yooseph S, Wu D, Eisen JA, Hoffman JM, Remington K, Beeson K, Tran B, Smith H, Baden-Tillson H, Stewart C, Thorpe J, Freeman J, Andrews-Pfannkoch C, Venter JE, Li K, Kravitz S, Heidelberg JF, Utterback T, Rogers Y-H, Falcón LI, Souza V, Bonilla-Rosso G, Eguiarte LE, Karl DM, Sathyendranath S, Platt T, Bermingham E, Gallardo V, Tamayo-Castillo G, Ferrari MR, Strausberg RL, Nealson K, Friedman R, Frazier M, Venter JC. 2007. The Sorcerer II Global Ocean Sampling expedition: northwest Atlantic through eastern tropical Pacific. PLoS Biol 5:e77. doi:10.1371/journal.pbio.0050077. 17355176 PMC1821060

[20] Sunagawa S, Coelho LP, Chaffron S, Kultima JR, Labadie K, Salazar G, Djahanschiri B, Zeller G, Mende DR, Alberti A, Cornejo-Castillo FM, Costea PI, Cruaud C, d’Ovidio F, Engelen S, Ferrera I, Gasol JM, Guidi L, Hildebrand F, Kokoszka F, Lepoivre C, Lima-Mendez G, Poulain J, Poulos BT, Royo-Llonch M, Sarmento H, Vieira-Silva S, Dimier C, Picheral M, Searson S, Kandels-Lewis S, Tara Oceans coordinators, Bowler C, de Vargas C, Gorsky G, Grimsley N, Hingamp P, Iudicone D, Jaillon O, Not F, Ogata H, Pesant S, Speich S, Stemmann L, Sullivan MB, Weissenbach J, Wincker P, Karsenti E, Raes J, Acinas SG, Bork P. 2015. Ocean plankton. Structure and function of the global ocean microbiome. Science 348:1261359. doi:10.1126/science.1261359. 25999513

[21] Haro-Moreno JM, López-Pérez M, de la Torre JR, Picazo A, Camacho A, Rodriguez-Valera F. 2018. Fine metagenomic profile of the Mediterranean stratified and mixed water columns revealed by assembly and recruitment. Microbiome 6:128. doi:10.1186/s40168-018-0513-5. 29991350 PMC6040077

[22] Pachiadaki MG, Brown JM, Brown J, Bezuidt O, Berube PM, Biller SJ, Poulton NJ, Burkart MD, La Clair JJ, Chisholm SW, Stepanauskas R. 2019. Charting the complexity of the marine microbiome through single-cell genomics. Cell 179:1623–1635.e11. doi:10.1016/j.cell.2019.11.017. 31835036 PMC6919566

[23] Levin PA, Angert ER. 2015. Small but mighty: cell size and bacteria. Cold Spring Harb Perspect Biol 7:a019216. doi:10.1101/cshperspect.a019216. 26054743 PMC4484965

[24] Kirchman DL. 2016. Growth rates of microbes in the oceans. Annu Rev Mar Sci 8:285–309. doi:10.1146/annurev-marine-122414-033938. 26195108

[25] Giovannoni SJ, Cameron Thrash J, Temperton B. 2014. Implications of streamlining theory for microbial ecology. ISME J 8:1553–1565. doi:10.1038/ismej.2014.60. 24739623 PMC4817614

[26] Rappé MS, Connon SA, Vergin KL, Giovannoni SJ. 2002. Cultivation of the ubiquitous SAR11 marine bacterioplankton clade. Nature 418:630–633. doi:10.1038/nature00917. 12167859

[27] Dupont CL, Rusch DB, Yooseph S, Lombardo MJ, Alexander Richter R, Valas R, Novotny M, Yee-Greenbaum J, Selengut JD, Haft DH, Halpern AL, Lasken RS, Nealson K, Friedman R, Craig Venter J. 2012. Genomic insights to SAR86, an abundant and uncultivated marine bacterial lineage. ISME J 6:1186–1199. doi:10.1038/ismej.2011.189. 22170421 PMC3358033

[28] Mizuno CM, Rodriguez-Valera F, Ghai R. 2015. Genomes of planktonic acidimicrobiales: widening horizons for marine actinobacteria by metagenomics. mBio 6:e02083-14. doi:10.1128/mBio.02083-14. 25670777 PMC4337565

[29] Giovannoni SJ, Rappé M. 2000. Evolution, diversity, and molecular ecology of marine prokaryotes, p 47–84. *In* Kirchman DL (ed), Microbial ecology of the oceans. John Wiley & Sons, Inc, New York, NY.

[30] Giovannoni SJ, Vergin KL. 2012. Seasonality in ocean microbial communities. Science 335:671–676. doi:10.1126/science.1198078. 22323811

[31] Oh HM, Kwon KK, Kang I, Kang SG, Lee JH, Kim SJ, Cho JC. 2010. Complete genome sequence of “*Candidatus* Puniceispirillum marinum” IMCC1322, a representative of the SAR116 clade in the Alphaproteobacteria. J Bacteriol 192:3240–3241. doi:10.1128/JB.00347-10. 20382761 PMC2901696

[32] Grote J, Bayindirli C, Bergauer K, Carpintero de Moraes P, Chen H, D’Ambrosio L, Edwards B, Fernández-Gómez B, Hamisi M, Logares R, Nguyen D, Rii YM, Saeck E, Schutte C, Widner B, Church MJ, Steward GF, Karl DM, Delong EF, Eppley JM, Schuster SC, Kyrpides NC, Rappé MS. 2011. Draft genome sequence of strain HIMB100, a cultured representative of the SAR116 clade of marine Alphaproteobacteria. Stand Genomic Sci 5:269–278. doi:10.4056/sigs.1854551. 22675578 PMC3368413

[33] Choi DH, Park KT, An SM, Lee K, Cho JC, Lee JH, Kim D, Jeon D, Noh JH. 2015. Pyrosequencing revealed SAR116 clade as dominant dddP-containing bacteria in oligotrophic NW Pacific Ocean. PLoS One 10:e0116271. doi:10.1371/journal.pone.0116271. 25615446 PMC4304780

[34] Sievert SM, Kiene RP, Schulz-Vogt HN. 2007. The sulfur cycle. Oceanography 20:117–123. doi:10.5670/oceanog.2007.55.

[35] Yoch DC. 2002. Dimethylsulfoniopropionate: its sources, role in the marine food web, and biological degradation to dimethylsulfide. Appl Environ Microbiol 68:5804–5815. doi:10.1128/AEM.68.12.5804-5815.2002. 12450799 PMC134419

[36] Bullock HA, Luo H, Whitman WB. 2017. Evolution of dimethylsulfoniopropionate metabolism in marine phytoplankton and bacteria. Front Microbiol 8:637. doi:10.3389/fmicb.2017.00637. 28469605 PMC5395565

[37] González JM, Hernández L, Manzano I, Pedrós-Alió C. 2019. Functional annotation of orthologs in metagenomes: a case study of genes for the transformation of oceanic dimethylsulfoniopropionate. ISME J 13:1183–1197. doi:10.1038/s41396-019-0347-6. 30643200 PMC6474240

[38] Parks DH, Rinke C, Chuvochina M, Chaumeil P-A, Woodcroft BJ, Evans PN, Hugenholtz P, Tyson GW. 2017. Recovery of nearly 8,000 metagenome-assembled genomes substantially expands the tree of life. Nat Microbiol 2:1533–1542. doi:10.1038/s41564-017-0012-7. 28894102

[39] Haroon MF, Thompson LR, Parks DH, Hugenholtz P, Stingl U. 2016. A catalogue of 136 microbial draft genomes from Red Sea metagenomes. Sci Data 3:160050. doi:10.1038/sdata.2016.50. 27377622 PMC4932879

[40] Tully BJ, Graham ED, Heidelberg JF. 2018. The reconstruction of 2,631 draft metagenome-assembled genomes from the global oceans. Sci Data 5:170203. doi:10.1038/sdata.2017.203. 29337314 PMC5769542

[41] Bowers RM, Kyrpides NC, Stepanauskas R, Harmon-Smith M, Doud D, Reddy TBK, Schulz F, Jarett J, Rivers AR, Eloe-Fadrosh EA, Tringe SG, Ivanova NN, Copeland A, Clum A, Becraft ED, Malmstrom RR, Birren B, Podar M, Bork P, Weinstock GM, Garrity GM, Dodsworth JA, Yooseph S, Sutton G, Glöckner FO, Gilbert JA, Nelson WC, Hallam SJ, Jungbluth SP, Ettema TJG, Tighe S, Konstantinidis KT, Liu WT, Baker BJ, Rattei T, Eisen JA, Hedlund B, McMahon KD, Fierer N, Knight R, Finn R, Cochrane G, Karsch-Mizrachi I, Tyson GW, Rinke C, Genome Standards Consortium, Lapidus A, Meyer F, Yilmaz P, Parks DH, Eren AM, Schriml L, Banfield JF, Hugenholtz P, Woyke T. 2017. Minimum information about a single amplified genome (MISAG) and a metagenome-assembled genome (MIMAG) of bacteria and archaea. Nat Biotechnol 35:725–731. doi:10.1038/nbt.3893. 28787424 PMC6436528

[42] Parks DH, Chuvochina M, Waite DW, Rinke C, Skarshewski A, Chaumeil P-A, Hugenholtz P. 2018. A standardized bacterial taxonomy based on genome phylogeny substantially revises the tree of life. Nat Biotechnol 36:996–1004. doi:10.1038/nbt.4229. 30148503

[43] Martinez-Gutierrez CA, Aylward FO. 2019. Strong purifying selection is associated with genome streamlining in epipelagic marinimicrobia. Genome Biol Evol 11:2887–2894. doi:10.1093/gbe/evz201. 31539038 PMC6798728

[44] López-Pérez M, Kimes NE, Haro-Moreno JM, Rodriguez-Valera F. 2016. Not all particles are equal: the selective enrichment of particle-associated bacteria from the Mediterranean Sea. Front Microbiol 7:996. doi:10.3389/fmicb.2016.00996. 27446036 PMC4916215

[45] López-Pérez M, Haro-Moreno JM, Coutinho FH, Martinez-Garcia M, Rodriguez-Valera F. 2020. The evolutionary success of the marine bacterium SAR11 analyzed through a metagenomic perspective. mSystems 5:e00605-20. doi:10.1128/mSystems.00605-20. 33024052 PMC7542561

[46] López-Pérez M, Haro-Moreno JM, Iranzo J, Rodriguez‐Valera F. 2020. Genomes of the “*Candidatus* Actinomarinales” order: highly streamlined marine epipelagic actinobacteria. mSystems 5:e01041-20. doi:10.1128/mSystems.01041-20. 33323418 PMC7771536

[47] Graham ED, Tully BJ. 2021. Marine Dadabacteria exhibit genome streamlining and phototrophy-driven niche partitioning. ISME J 15:1248–1256. doi:10.1038/s41396-020-00834-5. 33230264 PMC8115339

[48] Haro‐Moreno JM, Rodriguez‐Valera F, Rosselli R, Martinez‐Hernandez F, Roda‐Garcia JJ, Gomez ML, Fornas O, Martinez‐Garcia M, López‐Pérez M. 2020. Ecogenomics of the SAR11 clade. Environ Microbiol 22:1748–1763. doi:10.1111/1462-2920.14896. 31840364 PMC7318151

[49] Orellana LH, Ben Francis T, Krüger K, Teeling H, Müller MC, Fuchs BM, Konstantinidis KT, Amann RI. 2019. Niche differentiation among annually recurrent coastal Marine Group II Euryarchaeota. ISME J 13:3024–3036. doi:10.1038/s41396-019-0491-z. 31447484 PMC6864105

[50] Rodriguez-Valera F, Martin-Cuadrado A-B, Rodriguez-Brito B, Pasić L, Thingstad TF, Rohwer F, Mira A. 2009. Explaining microbial population genomics through phage predation. Nat Rev Microbiol 7:828–836. doi:10.1038/nrmicro2235. 19834481

[51] Reyes-Prieto A, Barquera B, Juárez O. 2014. Origin and evolution of the sodium-pumping NADH: ubiquinone oxidoreductase. PLoS One 9:e96696. doi:10.1371/journal.pone.0096696. 24809444 PMC4014512

[52] Zhang H, Yoshizawa S, Sun Y, Huang Y, Chu X, González JM, Pinhassi J, Luo H. 2019. Repeated evolutionary transitions of flavobacteria from marine to non-marine habitats. Environ Microbiol 21:648–666. doi:10.1111/1462-2920.14509. 30565818

[53] Getz EW, Tithi SS, Zhang L, Aylward FO. 2018. Parallel evolution of genome streamlining and cellular bioenergetics across the marine radiation of a bacterial phylum. mBio 9:e01089-18. doi:10.1128/mBio.01089-18. 30228235 PMC6143742

[54] Salcher MM, Schaefle D, Kaspar M, Neuenschwander SM, Ghai R. 2019. Evolution in action: habitat transition from sediment to the pelagial leads to genome streamlining in Methylophilaceae. ISME J 13:2764–2777. doi:10.1038/s41396-019-0471-3. 31292537 PMC6794327

[55] Kim AD, Baker AS, Dunaway-Mariano D, Metcalf WW, Wanner BL, Martin BM. 2002. The 2-aminoethylphosphonate-specific transaminase of the 2-aminoethylphosphonate degradation pathway. J Bacteriol 184:4134–4140. doi:10.1128/JB.184.15.4134-4140.2002. 12107130 PMC135204

[56] Villarreal-Chiu J, Quinn J, McGrath J. 2012. The genes and enzymes of phosphonate metabolism by bacteria, and their distribution in the marine environment. Front Microbiol 3:19. doi:10.3389/fmicb.2012.00019. 22303297 PMC3266647

[57] Sowell SM, Wilhelm LJ, Norbeck AD, Lipton MS, Nicora CD, Barofsky DF, Carlson CA, Smith RD, Giovanonni SJ. 2009. Transport functions dominate the SAR11 metaproteome at low-nutrient extremes in the Sargasso Sea. ISME J 3:93–105. doi:10.1038/ismej.2008.83. 18769456

[58] Béjà O, Suzuki MT, Hadd A, Nguyen LP, Spudich JL, Spudich EN, Delong EF. 2000. Bacterial rhodopsin: evidence for a new type of phototrophy in the sea. Science 289:1902–1906. doi:10.1126/science.289.5486.1902. 10988064

[59] Olson DK, Yoshizawa S, Boeuf D, Iwasaki W, DeLong EF. 2018. Proteorhodopsin variability and distribution in the North Pacific Subtropical Gyre. ISME J 12:1047–1060. doi:10.1038/s41396-018-0074-4. 29476140 PMC5864233

[60] Man D, Wang W, Sabehi G, Aravind L, Post AF, Massana R, Spudich EN, Spudich JL, Béjà O. 2003. Diversification and spectral tuning in marine proteorhodopsins. EMBO J 22:1725–1731. doi:10.1093/emboj/cdg183. 12682005 PMC154475

[61] Morris RM, Rappé MS, Connon SA, Vergin KL, Siebold WA, Carlson CA, Giovannoni SJ. 2002. SAR11 clade dominates ocean surface bacterioplankton communities. Nature 420:806–810. doi:10.1038/nature01240. 12490947

[62] Nakajima Y, Kojima K, Kashiyama Y, Doi S, Nakai R, Sudo Y, Kogure K, Yoshizawa S. 2020. Bacterium lacking a known gene for retinal biosynthesis constructs functional rhodopsins. Microbes Environ 35 (4). doi:10.1264/jsme2.ME20085. PMC7734400 33281127

[63] Kappler U, Schäfer H. 2014. Transformations of dimethylsulfide, p 279–313. In Kroneck PMH, Sosa Torres ME (ed), The metal-driven biogeochemistry of gaseous compounds in the environment. Springer, Dordrecht, Netherlands.

[64] Reisch CR, Stoudemayer MJ, Varaljay VA, Amster IJ, Moran MA, Whitman WB. 2011. Novel pathway for assimilation of dimethylsulphoniopropionate widespread in marine bacteria. Nature 473:208–211. doi:10.1038/nature10078. 21562561

[65] Shah V, Zhao X, Lundeen RA, Ingalls AE, Nicastro D, Morris RM. 2019. Morphological plasticity in a sulfur-oxidizing marine bacterium from the SUP05 clade enhances dark carbon fixation. mBio 10:e00216-19. doi:10.1128/mBio.00216-19. 31064824 PMC6509183

[66] Savoie ER, Lanclos VC, Henson MW, Cheng C, Getz EW, Barnes SJ, LaRowe DE, Rappé MS, Thrash JC. 2021. Ecophysiology of the cosmopolitan OM252 bacterioplankton (Gammaproteobacteria). mSystems 6:e00276-21. doi:10.1128/mSystems.00276-21. PMC8269220 34184914

[67] Moran MA, Buchan A, González JM, Heidelberg JF, Whitman WB, Kiene RP, Henriksen JR, King GM, Belas R, Fuqua C, Brinkac L, Lewis M, Johri S, Weaver B, Pai G, Eisen JA, Rahe E, Sheldon WM, Ye W, Miller TR, Carlton J, Rasko DA, Paulsen IT, Ren Q, Daugherty SC, Deboy RT, Dodson RJ, Durkin AS, Madupu R, Nelson WC, Sullivan SA, Rosovitz MJ, Haft DH, Selengut J, Ward N. 2004. Genome sequence of Silicibacter pomeroyi reveals adaptations to the marine environment. Nature 432:910–913. doi:10.1038/nature03170. 15602564

[68] Poretsky RS, Hewson I, Sun S, Allen AE, Zehr JP, Moran MA. 2009. Comparative day/night metatranscriptomic analysis of microbial communities in the North Pacific subtropical gyre. Environ Microbiol 11:1358–1375. doi:10.1111/j.1462-2920.2008.01863.x. 19207571

[69] Tuttle JH, Jannasch HW. 1977. Thiosulfate stimulation of microbial dark assimilation of carbon dioxide in shallow marine waters. Microb Ecol 4:9–25. doi:10.1007/BF02010426. 24231882

[70] Ghosh W, Dam B. 2009. Biochemistry and molecular biology of lithotrophic sulfur oxidation by taxonomically and ecologically diverse bacteria and archaea. FEMS Microbiol Rev 33:999–1043. doi:10.1111/j.1574-6976.2009.00187.x. 19645821

[71] van Vliet DM, von Meijenfeldt FAB, Dutilh BE, Villanueva L, Sinninghe Damsté JS, Stams AJM, Sánchez‐Andrea I. 2021. The bacterial sulfur cycle in expanding dysoxic and euxinic marine waters. Environ Microbiol 23:2834–2857. doi:10.1111/1462-2920.15265. 33000514 PMC8359478

[72] Giovannoni SJ. 2017. SAR11 bacteria: the most abundant plankton in the oceans. Annu Rev Mar Sci 9:231–255. doi:10.1146/annurev-marine-010814-015934. 27687974

[73] Haro-Moreno JM, López-Pérez M, Rodríguez-Valera F. 2021. Enhanced recovery of microbial genes and genomes from a marine water column using long-read metagenomics. Front Microbiol 12:2410. doi:10.3389/fmicb.2021.708782. PMC8430335 34512586

[74] Zhang Y, Sun Y, Jiao N, Stepanauskas R, Luo H. 2016. Ecological genomics of the uncultivated marine Roseobacter lineage CHAB-I-5. Appl Environ Microbiol 82:2100–2111. doi:10.1128/AEM.03678-15. 26826224 PMC4807517

[75] Parks DH, Imelfort M, Skennerton CT, Hugenholtz P, Tyson GW. 2015. CheckM: assessing the quality of microbial genomes recovered from isolates, single cells, and metagenomes. Genome Res 25:1043–1055. doi:10.1101/gr.186072.114. 25977477 PMC4484387

[76] Segata N, Börnigen D, Morgan XC, Huttenhower C. 2013. PhyloPhlAn is a new method for improved phylogenetic and taxonomic placement of microbes. Nat Commun 4:2304. doi:10.1038/ncomms3304. 23942190 PMC3760377

[77] Nguyen LT, Schmidt HA, Von Haeseler A, Minh BQ. 2015. IQ-TREE: a fast and effective stochastic algorithm for estimating maximum-likelihood phylogenies. Mol Biol Evol 32:268–274. doi:10.1093/molbev/msu300. 25371430 PMC4271533

[78] Hoang DT, Chernomor O, von Haeseler A, Minh BQ, Vinh LS. 2018. UFBoot2: improving the ultrafast bootstrap approximation. Mol Biol Evol 35:518–522. doi:10.1093/molbev/msx281. 29077904 PMC5850222

[79] Letunic I, Bork P. 2016. Interactive tree of life (iTOL) v3: an online tool for the display and annotation of phylogenetic and other trees. Nucleic Acids Res 44:W242–W245. doi:10.1093/nar/gkw290. 27095192 PMC4987883

[80] Hyatt D, Chen G-L, Locascio PF, Land ML, Larimer FW, Hauser LJ. 2010. Prodigal: prokaryotic gene recognition and translation initiation site identification. BMC Bioinformatics 11:119. doi:10.1186/1471-2105-11-119. 20211023 PMC2848648

[81] Buchfink B, Xie C, Huson DH. 2015. Fast and sensitive protein alignment using DIAMOND. Nat Methods 12:59–60. doi:10.1038/nmeth.3176. 25402007

[82] Tatusov RL, Natale DA, Garkavtsev IV, Tatusova TA, Shankavaram UT, Rao BS, Kiryutin B, Galperin MY, Fedorova ND, Koonin EV. 2001. The COG database: new developments in phylogenetic classification of proteins from complete genomes. Nucleic Acids Res 29:22–28. doi:10.1093/nar/29.1.22. 11125040 PMC29819

[83] Haft DH, Loftus BJ, Richardson DL, Yang F, Eisen JA, Paulsen IT, White O. 2001. TIGRFAMs: a protein family resource for the functional identification of proteins. Nucleic Acids Res 29:41–43. doi:10.1093/nar/29.1.41. 11125044 PMC29844

[84] Eddy SR. 2011. Accelerated profile HMM searches. PLoS Comput Biol 7:e1002195. doi:10.1371/journal.pcbi.1002195. 22039361 PMC3197634

[85] Lowe TM, Eddy SR. 1997. tRNAscan-SE: a program for improved detection of transfer RNA genes in genomic sequence. Nucleic Acids Res 25:955–964. doi:10.1093/nar/25.5.955. 9023104 PMC146525

[86] Nawrocki EP. 2009. Structural RNA homology search and alignment using covariance models. PhD dissertation. Washington University in St. Louis, St. Louis, MO.

[87] Huang Y, Gilna P, Li W. 2009. Identification of ribosomal RNA genes in metagenomic fragments. Bioinformatics 25:1338–1340. doi:10.1093/bioinformatics/btp161. 19346323 PMC2677747

[88] Richter M, Rossello-Mora R. 2009. Shifting the genomic gold standard for the prokaryotic species definition. Proc Natl Acad Sci USA 106:19126–19131. doi:10.1073/pnas.0906412106. 19855009 PMC2776425

[89] Rodriguez-R LM, Konstantinidis KT. 2016. The enveomics collection: a toolbox for specialized analyses of microbial genomes and metagenomes. PeerJ Prepr doi:10.7287/peerj.preprints.1900v1.

[90] Rice P, Longden I, Bleasby A. 2000. EMBOSS: The European Molecular Biology Open Software Suite. Trends Genet 16:276–277. doi:10.1016/s0168-9525(00)02024-2. 10827456

[91] Huang Y, Niu B, Gao Y, Fu L, Li W. 2010. CD-HIT suite: a web server for clustering and comparing biological sequences. Bioinformatics 26:680–682. doi:10.1093/bioinformatics/btq003. 20053844 PMC2828112

[92] Mende DR, Bryant JA, Aylward FO, Eppley JM, Nielsen T, Karl DM, DeLong EF. 2017. Environmental drivers of a microbial genomic transition zone in the ocean’s interior. Nat Microbiol 2:1367–1373. doi:10.1038/s41564-017-0008-3. 28808230

[93] Stingl U, Tripp HJ, Giovannoni SJ. 2007. Improvements of high-throughput culturing yielded novel SAR11 strains and other abundant marine bacteria from the Oregon coast and the Bermuda Atlantic Time Series study site. ISME J 1:361–371. doi:10.1038/ismej.2007.49. 18043647

[94] Gonzaga A, López-Pérez A, Martin-Cuadrado AB, Ghai R, Rodriguez-Valera F. 2012. Complete genome sequence of the copiotrophic marine bacterium Alteromonas macleodii strain ATCC 27126T. J Bacteriol 194:6998. doi:10.1128/JB.01565-12. 23209244 PMC3510622

[95] Niero H, da Silva MAC, de Felicio R, Trivella DBB, de Lima AOS. 2021. Carotenoids produced by the deep-sea bacterium Erythrobacter citreus LAMA 915: detection and proposal of their biosynthetic pathway. Folia Microbiol (Praha) 66:441–456. doi:10.1007/s12223-021-00858-0. 33723710

[96] Sanfilippo JE, Nguyen AA, Garczarek L, Karty JA, Pokhrel S, Strnat JA, Partensky F, Schluchter WM, Kehoe DM. 2019. Interplay between differentially expressed enzymes contributes to light color acclimation in marine Synechococcus. Proc Natl Acad Sci USA 116:6457–6462. doi:10.1073/pnas.1810491116. 30846551 PMC6442610

[97] Santoro AE, Dupont CL, Richter RA, Craig MT, Carini P, McIlvin MR, Yang Y, Orsi WD, Moran DM, Saito MA. 2015. Genomic and proteomic characterization of “*Candidatus* Nitrosopelagicus brevis”: an ammonia-oxidizing archaeon from the open ocean. Proc Natl Acad Sci USA 112:1173–1178. doi:10.1073/pnas.1416223112. 25587132 PMC4313803

[98] Dufresne A, Salanoubat M, Partensky F, Artiguenave F, Axmann IM, Barbe V, Duprat S, Galperin MY, Koonin EV, Le Gall F, Makarova KS, Ostrowski M, Oztas S, Robert C, Rogozin IB, Scanlan DJ, de Marsac NT, Weissenbach J, Wincker P, Wolf YI, Hess WR. 2003. Genome sequence of the cyanobacterium Prochlorococcus marinus SS120, a nearly minimal oxyphototrophic genome. Proc Natl Acad Sci USA 100:10020–10025. doi:10.1073/pnas.1733211100. 12917486 PMC187748

[99] Blattner FR, Plunkett G, Bloch CA, Perna NT, Burland V, Riley M, Collado-Vides J, Glasner JD, Rode CK, Mayhew GF, Gregor J, Davis NW, Kirkpatrick HA, Goeden MA, Rose DJ, Mau B, Shao Y. 1997. The complete genome sequence of Escherichia coli K-12. Science 277:1453–1462. doi:10.1126/science.277.5331.1453. 9278503

[100] Drost H-G, Gabel A, Grosse I, Quint M. 2015. Evidence for active maintenance of phylotranscriptomic hourglass patterns in animal and plant embryogenesis. Mol Biol Evol 32:1221–1231. doi:10.1093/molbev/msv012. 25631928 PMC4408408

[101] Suyama M, Torrents D, Bork P. 2006. PAL2NAL: robust conversion of protein sequence alignments into the corresponding codon alignments. Nucleic Acids Res 34:W609–W612. doi:10.1093/nar/gkl315. 16845082 PMC1538804

[102] Yang Z, Nielsen R. 2000. Estimating synonymous and nonsynonymous substitution rates under realistic evolutionary models. Mol Biol Evol 17:32–43. doi:10.1093/oxfordjournals.molbev.a026236. 10666704

[103] Lê S, Josse J, Husson F. 2008. FactoMineR: an R package for multivariate analysis. J Stat Softw 25:1–18.

[104] Altschul SF, Madden TL, Schäffer AA, Zhang J, Zhang Z, Miller W, Lipman DJ. 1997. Gapped BLAST and PSI-BLAST: a new generation of protein database search programs. Nucleic Acids Res 25:3389–3402. doi:10.1093/nar/25.17.3389. 9254694 PMC146917

[105] Lombard V, Golaconda Ramulu H, Drula E, Coutinho PM, Henrissat B. 2014. The carbohydrate-active enzymes database (CAZy) in 2013. Nucleic Acids Res 42:D490–D495. doi:10.1093/nar/gkt1178. 24270786 PMC3965031

[106] Yin Y, Mao X, Yang J, Chen X, Mao F, Xu Y. 2012. DbCAN: a web resource for automated carbohydrate-active enzyme annotation. Nucleic Acids Res 40:W445–W451. doi:10.1093/nar/gks479. 22645317 PMC3394287

[107] Kanehisa M, Sato Y, Kawashima M, Furumichi M, Tanabe M. 2016. KEGG as a reference resource for gene and protein annotation. Nucleic Acids Res 44:D457–D462. doi:10.1093/nar/gkv1070. 26476454 PMC4702792

[108] Kanehisa M, Sato Y, Morishima K. 2016. BlastKOALA and GhostKOALA: KEGG tools for functional characterization of genome and metagenome sequences. J Mol Biol 428:726–731. doi:10.1016/j.jmb.2015.11.006. 26585406

[109] Caspi R, Billington R, Keseler IM, Kothari A, Krummenacker M, Midford PE, Ong WK, Paley S, Subhraveti P, Karp PD. 2020. The MetaCyc database of metabolic pathways and enzymes - a 2019 update. Nucleic Acids Res 48:D445–D453. doi:10.1093/nar/gkz862. 31586394 PMC6943030

