# Supplementary figures and images for: Phylogenomics of SAR116 Clade Reveals Two Subclades with Different Evolutionary Trajectories and an Important Role in the Ocean Sulfur Cycle
Source: mSystems. 2021 Oct 5;6(5):e00944-21. doi: 10.1128/mSystems.00944-21 (PMC8547437; doi:10.1128/mSystems.00944-21)

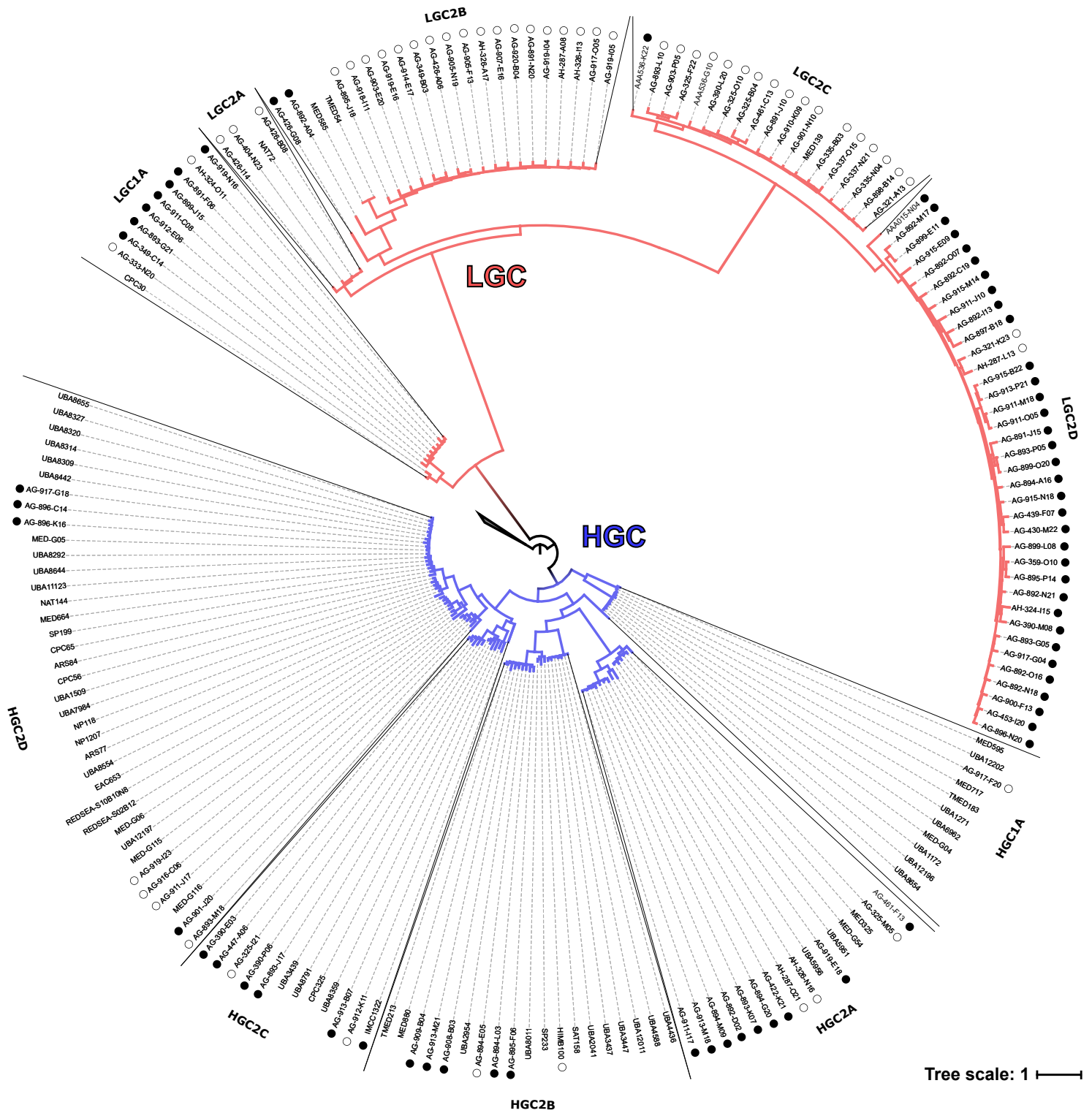

Supplement: FIG S1 [file msystems.00944-21-sf001.pdf]
