# Supplementary material for: Phylogenomics of SAR116 Clade Reveals Two Subclades with Different Evolutionary Trajectories and an Important Role in the Ocean Sulfur Cycle
Source: mSystems. 2021 Oct 5;6(5):e00944-21. doi: 10.1128/mSystems.00944-21 (PMC8547437; doi:10.1128/mSystems.00944-21)
Supplement: FIG S3 [file msystems.00944-21-sf003.pdf]

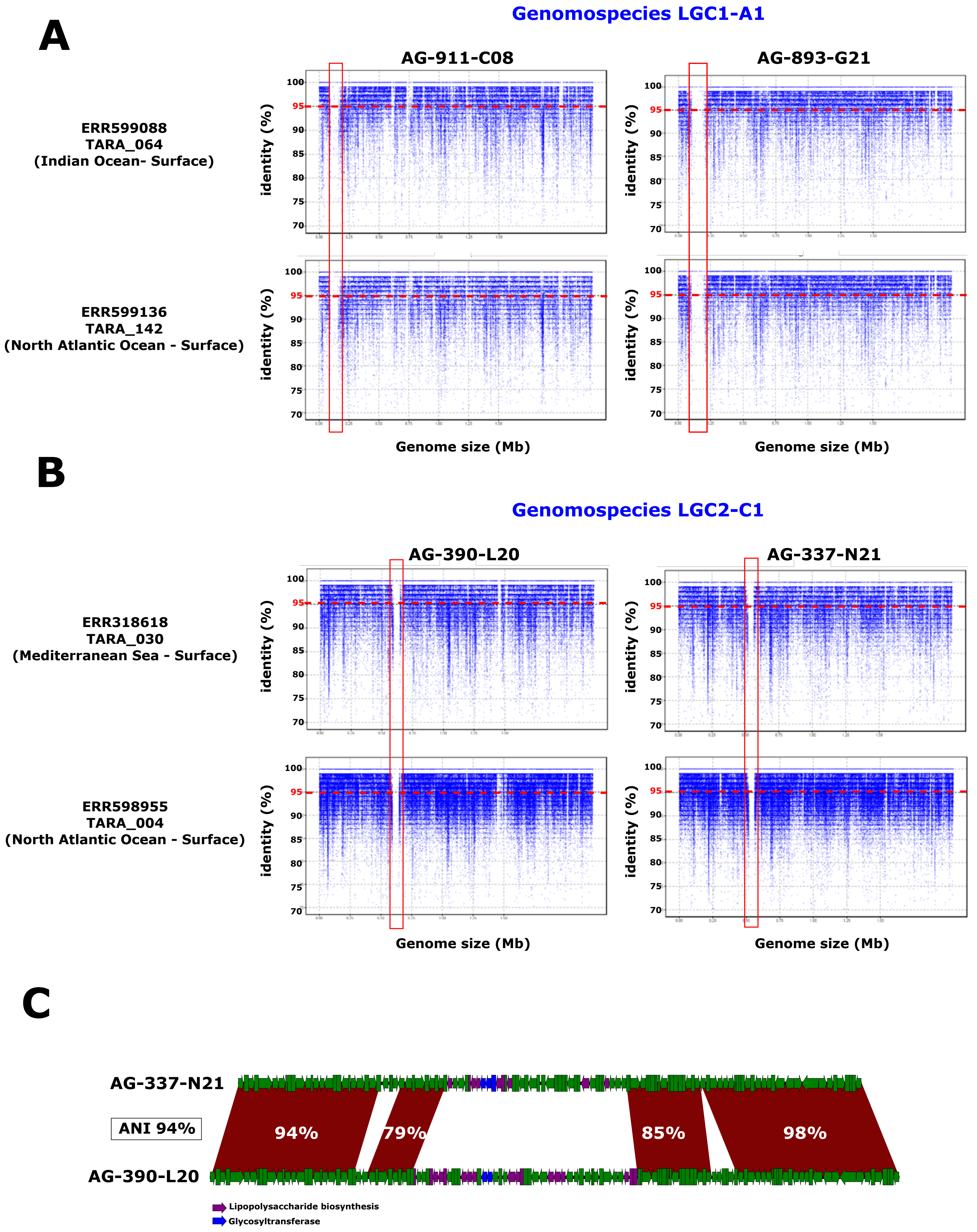
A
Genomospecies LGC1-A1
AG-911-C08
AG-893-G21
ERR599088
TARA_064
(Indian Ocean- Surface)
ERR599136
TARA_142
(North Atlantic Ocean - Surface)
identity (%)
Genome size (Mb)
B
Genomospecies LGC2-C1
AG-390-L20
AG-337-N21
ERR318618
TARA_030
(Mediterranean Sea - Surface)
ERR598955
TARA_004
(North Atlantic Ocean - Surface)
identity (%)
Genome size (Mb)
C
AG-337-N21
ANI 94%
94%
79%
85%
98%
AG-390-L20
Lipopolysaccharide biosynthesis
Glycosyltransferase
