# Supplementary material for: Phylogenomics of SAR116 Clade Reveals Two Subclades with Different Evolutionary Trajectories and an Important Role in the Ocean Sulfur Cycle
Source: mSystems. 2021 Oct 5;6(5):e00944-21. doi: 10.1128/mSystems.00944-21 (PMC8547437; doi:10.1128/mSystems.00944-21)
Supplement: FIG S2 [file msystems.00944-21-sf002.pdf]

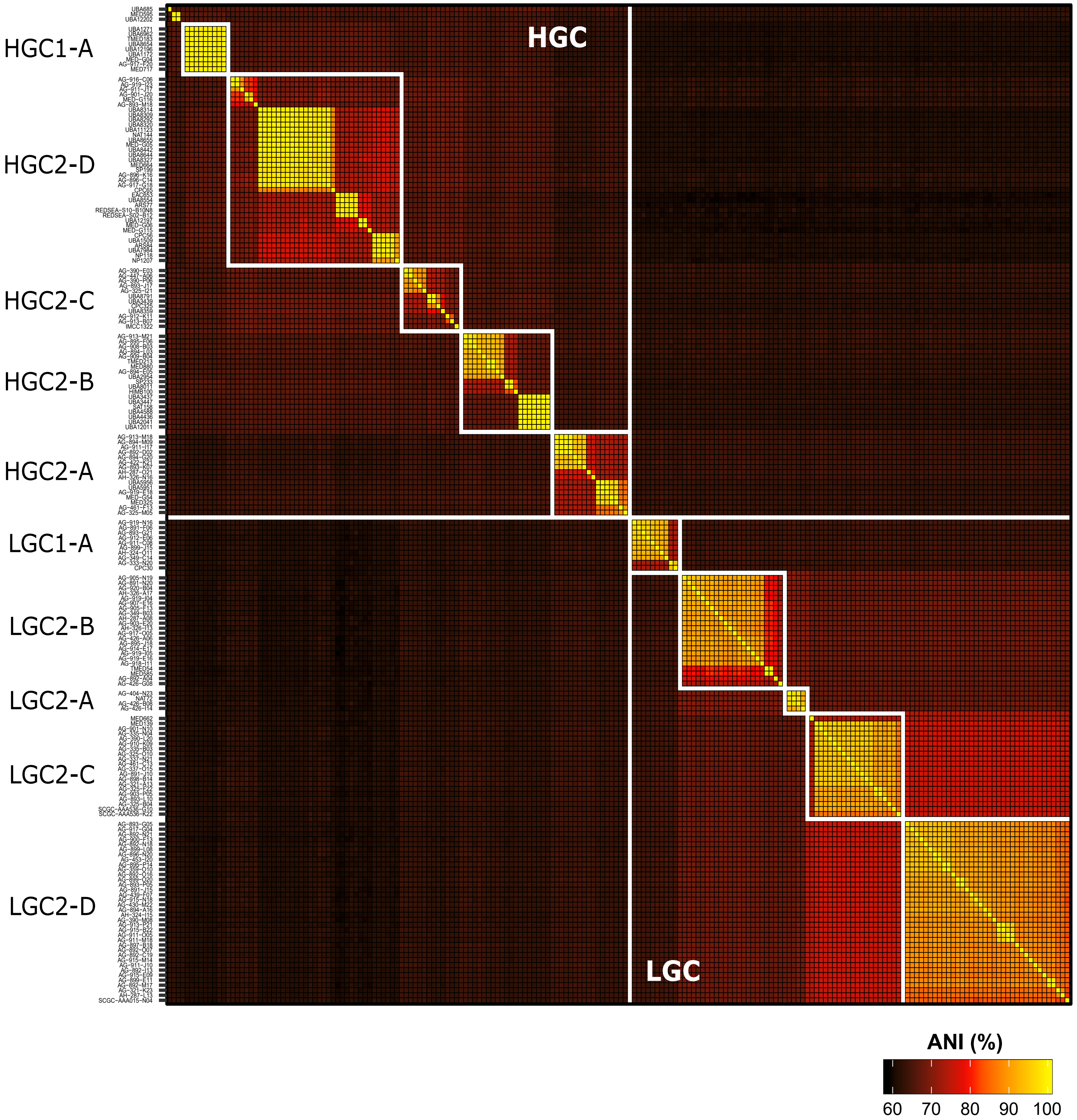
HGC1-A
HGC2-D
HGC2-C
HGC2-B
HGC2-A
LGC1-A
LGC2-B
LGC2-A
LGC2-C
LGC2-D
HGC
LGC
ANI (%)
60
70
80
90
100
