# Supplementary material for: Phylogenomics of SAR116 Clade Reveals Two Subclades with Different Evolutionary Trajectories and an Important Role in the Ocean Sulfur Cycle
Source: mSystems. 2021 Oct 5;6(5):e00944-21. doi: 10.1128/mSystems.00944-21 (PMC8547437; doi:10.1128/mSystems.00944-21)
Supplement: FIG S4 [file msystems.00944-21-sf004.pdf]

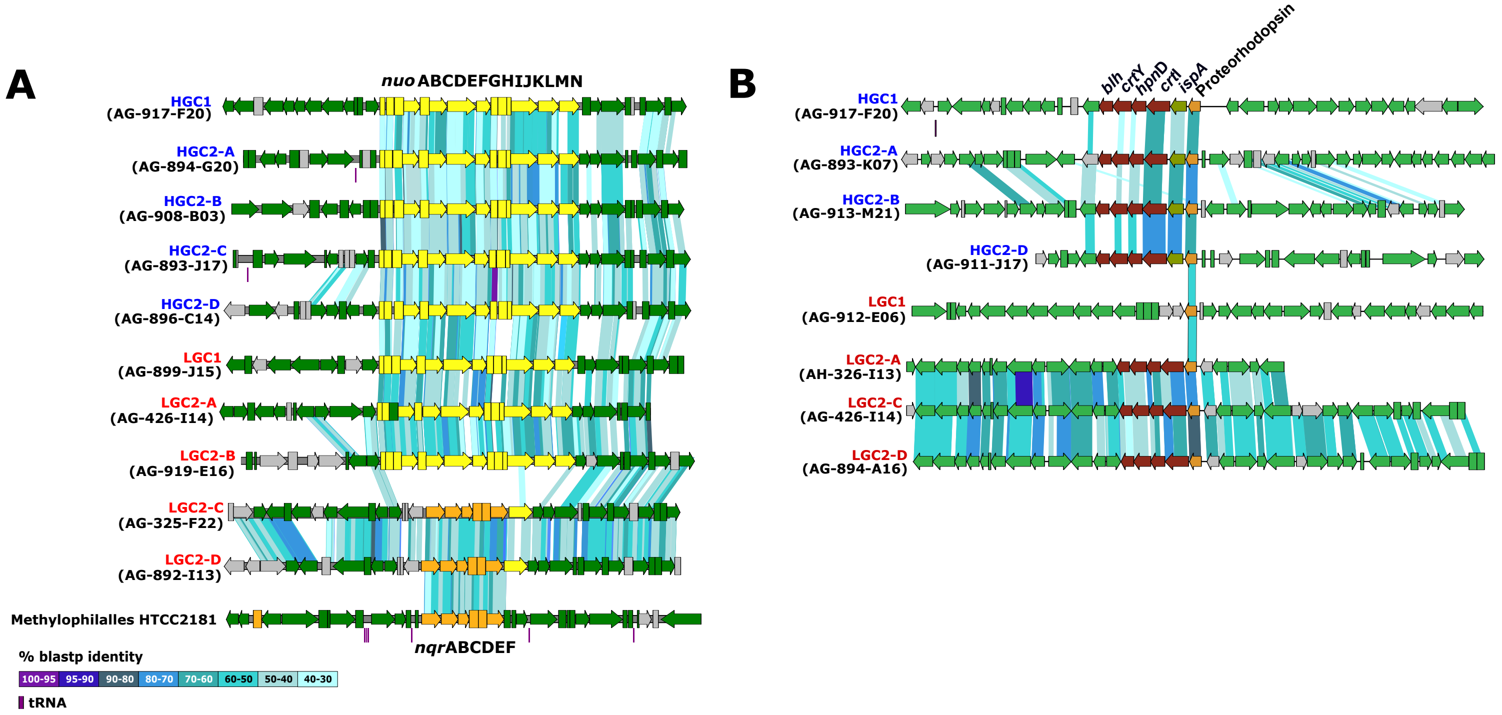
A
nuo ABCDEFGHIJKLMN
HGC1
(AG-917-F20)
HGC2-A
(AG-894-G20)
HGC2-B
(AG-908-B03)
HGC2-C
(AG-893-J17)
HGC2-D
(AG-896-C14)
LGC1
(AG-899-J15)
LGC2-A
(AG-426-I14)
LGC2-B
(AG-919-E16)
LGC2-C
(AG-325-F22)
LGC2-D
(AG-892-I13)
Methylophilalles HTCC2181
nqrABCDEF
% blastp identity
100-95
95-90
90-80
80-70
70-60
60-50
50-40
40-30
tRNA
B
blh
crtY
hpnD
crtI
ispA
Proteorhodopsin
HGC1
(AG-917-F20)
HGC2-A
(AG-893-K07)
HGC2-B
(AG-913-M21)
HGC2-D
(AG-911-J17)
LGC1
(AG-912-E06)
LGC2-A
(AH-326-I13)
LGC2-C
(AG-426-I14)
LGC2-D
(AG-894-A16)
